# Climate Change Impacts on Urban Sanitation: A Systematic Review and Failure Mode Analysis

**DOI:** 10.1021/acs.est.1c07424

**Published:** 2022-04-12

**Authors:** Leonie Hyde-Smith, Zhe Zhan, Katy Roelich, Anna Mdee, Barbara Evans

**Affiliations:** University of Leeds, Woodhouse Lane, Leeds LS2 9JT, U.K.

**Keywords:** extreme weather, sewer, CSO, combined sewer overflow, emptying, FSM, flood

## Abstract

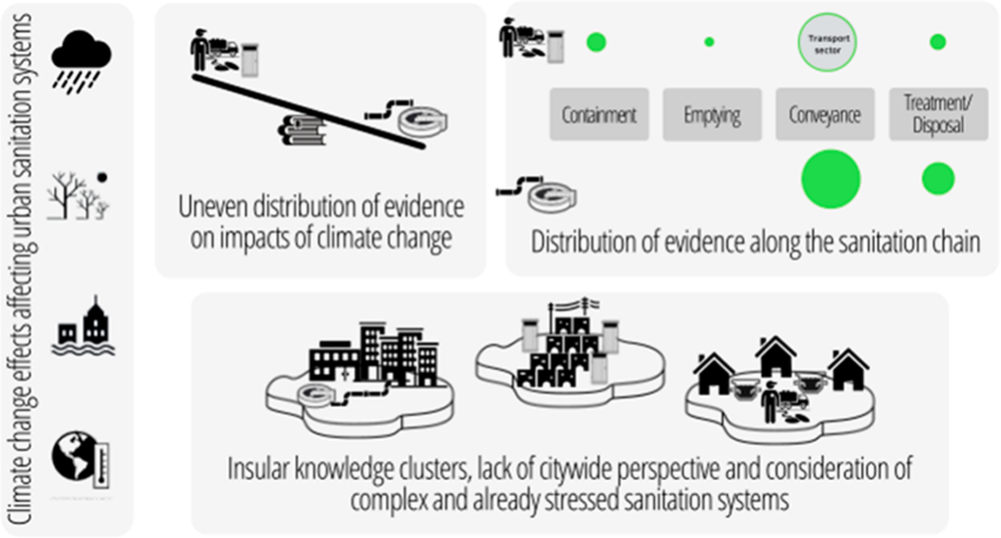

Climate change will stress urban sanitation systems. Although urban sanitation uses various infrastructure types and service systems, current research appears skewed toward a small subset of cases. We conducted a systematic literature review to critically appraise the evidence for climate change impacts on all urban sanitation system types. We included road-based transport networks, an essential part of fecal sludge management systems. We combined the evidence on climate change impacts with the existing knowledge about modes of urban sanitation failures. We found a predominance of studies that assess climate impacts on centralized sewerage in high-income contexts. The implications of climate change for urban nonsewered and complex, fragmented, and (partially) decentralized sanitation systems remain under-researched. In addition, the understanding of the impacts of climate change on urban sanitation systems fails to take a comprehensive citywide perspective considering interdependencies with other sectors and combinations of climate effects. We conclude that the evidence for climate change impacts on urban sanitation systems is weak. To date, research neither adequately represents the variety of urban sanitation infrastructure and service systems nor reflects the operational and management challenges of already stressed systems.

## Introduction

Effective sanitation systems are crucial for public and environmental health, particularly in densely populated urban areas where the risks from unsafe excreta disposal are compounded because of excreta volumes and the probability of exposure.^1^ Globally, around 1.9 billion people lack access to basic sanitation; more than a third of urban dwellers lack access to safely managed sanitation systems.^2^ The effects of climate change (CC) can damage or destroy sanitation infrastructure, disrupt services, and inhibit the system’s efficacy;^3−5^ CC will make achieving universal access to safely managed sanitation more expensive and slower.^6^

There have been a small number of reviews on the impacts of CC on urban sanitation. Primarily these have focused on changing precipitation patterns and consequent flood risks for cities relying on sewer-based urban waste- and stormwater management^7−9^ or included a broader overview on potential CC effects and impacts but also focused on sewerage.^5^

The first comprehensive attempts to identify potential impacts of CC on various sanitation systems and consider their vulnerability and resilience in low and lower-middle income countries (LMICs), were the Vision 2030 research commissioned by WHO^10−13^ and a scoping study led by the Overseas Development Institute on climate impacts on water resources and WASH systems.^14^ Since then, scholars have examined the resilience and adaptability of different sanitation technologies^15−17^ and applied the Vision 2030 sanitation resilience categories to specific countries.^18^ Recent summaries have incorporated the mentioned studies^19−21^ and provided valuable systems analysis on the impacts of flooding.^22^ Several international agencies have published guidelines and summary papers.^23−25^

However, there has not been a comprehensive summary and assessment of the evidence base for the likely impacts of CC on the range of urban sanitation systems or components of such systems generally found in low- and middle-income countries and high-income countries (HIC) contexts, integrating the evidence for impacts on sewered and nonsewered sanitation and highlighting the gaps in knowledge and rigor of assessment of CC impacts along the entire sanitation chain.

This study responds to this gap by delivering a systematic review that overlays knowledge about the failures of urban sanitation systems today with the stresses that a future climate will impose. We based our analysis on a recent review of urban sanitation in 39 cities, which articulates typical urban sanitation systems and corresponding failure modes based on the analysis of excreta flow diagrams.^26^ We used these failure modes to explore how CC may increase pressure on existing diverse sanitation systems, including household and city-scale infrastructure and services, which are often incomplete or poorly functioning.^26,27^

### Terminology and Framing

Locally the effects of global CC will be (or are already) felt as more intense or prolonged precipitation, more frequent or more intense storms or cyclones, more variable or declining rainfall or runoff, sea-level rise, or more variable and increasing temperatures, including temperature extremes. These CC effects can cause or exacerbate hazards or changes such as flooding, erosion, or changes in ground and surface water levels that directly impact sanitation systems.^25^ We categorize the potential impacts of CC on urban sanitation systems as follows:

- Negative direct impacts: (i) damaged sanitation infrastructure, (ii) disrupted services, and (iii) inhibited system efficacy.
- Positive direct impacts: (i) prolonged life or reduced maintenance requirements of infrastructure, (ii) improved service delivery (less disrupted emptying services), and (iii) improved system performance

Urban sanitation systems use a wide range of infrastructure, technologies, and service arrangements. Homogeneous systems using centralized sewerage and treatment are concentrated in HICs. Cities in LMICs are characterized by complex and (partially) decentralized and fragmented systems dominated by (nonsewered) fecal sludge management (FSM).^28−30^ These typically rely on onsite containment with manual or mechanical emptying and road-based conveyance of fecal sludge (FS) to a treatment facility. Most of these systems are designed to allow infiltration of the supernatant into the ground (soil-based treatment). However, in dense urban settlements, these systems are frequently poorly designed and constructed, resulting in inadequate supernatant treatment.^31^ Nonsewered systems account for most sanitation users globally and most urban dwellers in Central and Southern Asia, Oceania, and sub-Saharan Africa.^2^

### Review Question and Objectives

The review question was “What is the evidence for the impacts of climate change on urban sanitation systems?” The objectives were to (1) identify studies that assess or report on the impacts of CC on urban sanitation systems and rate the strength of this evidence; (2) analyze how the current understanding of the impacts of CC on urban sanitation systems relates to the knowledge about modes of urban sanitation failures;^26^ (3) identify gaps in the evidence of climate-related impacts in the context of complex urban sanitation systems. The review was registered on PROSPERO (CRD42021237370). Methods and findings are reported following the preferred reporting items for systematic reviews and meta-analysis (PRISMA).^32^

## Materials and Methods

### Literature Review

We conducted a systematic search in compliance with PRISMA guidelines^32^ to identify original qualitative or quantitative research on the impacts of CC on urban sanitation systems.

The review populations were systems or their components typically part of urban sanitation provision, including infrastructure or services. We excluded systems that only operate on a stand-alone basis or at household scale (often associated with rural areas). We also excluded urban drainage systems that are exclusively used for stormwater.

Outcomes of interest were categorized as potential and actual direct CC-related impacts on urban sanitation systems that affect the delivery of safely managed sanitation as defined in the introductory section.

The review was restricted to studies in English (original or translated) with no limits to the publishing date of the included literature. The authors included evidence published in peer-reviewed journal articles, published conference proceedings, and gray literature.

Table S1 provides a comprehensive list of inclusion and exclusion criteria.

Peer-reviewed literature was searched for a combination of three main concepts: climate change, sanitation systems, and impacts. As part of the sanitation system, we included road-based transport networks, arguing that they are as crucial to FSM as functional sewers are to wastewater conveyance. The search strategy for this review reflected this argument by including keywords for road-based transport networks as part of the sanitation systems. We included studies that made no explicit connection to CC but presented evidence on impacts on sanitation systems related to hazards (e.g., flooding, saline intrusion) that are likely to be exacerbated by CC. However, we excluded studies referring to the impact of “normal” weather variations (e.g., seasonal or daily variations) on sanitation systems. The search was conducted in February 2021 using electronic databases Scopus, Web of Science, Transport Database (OvidSP interface), and Global Health (OvidSP interface). Table 1 shows the search strategy for the database search.

**Table 1:**
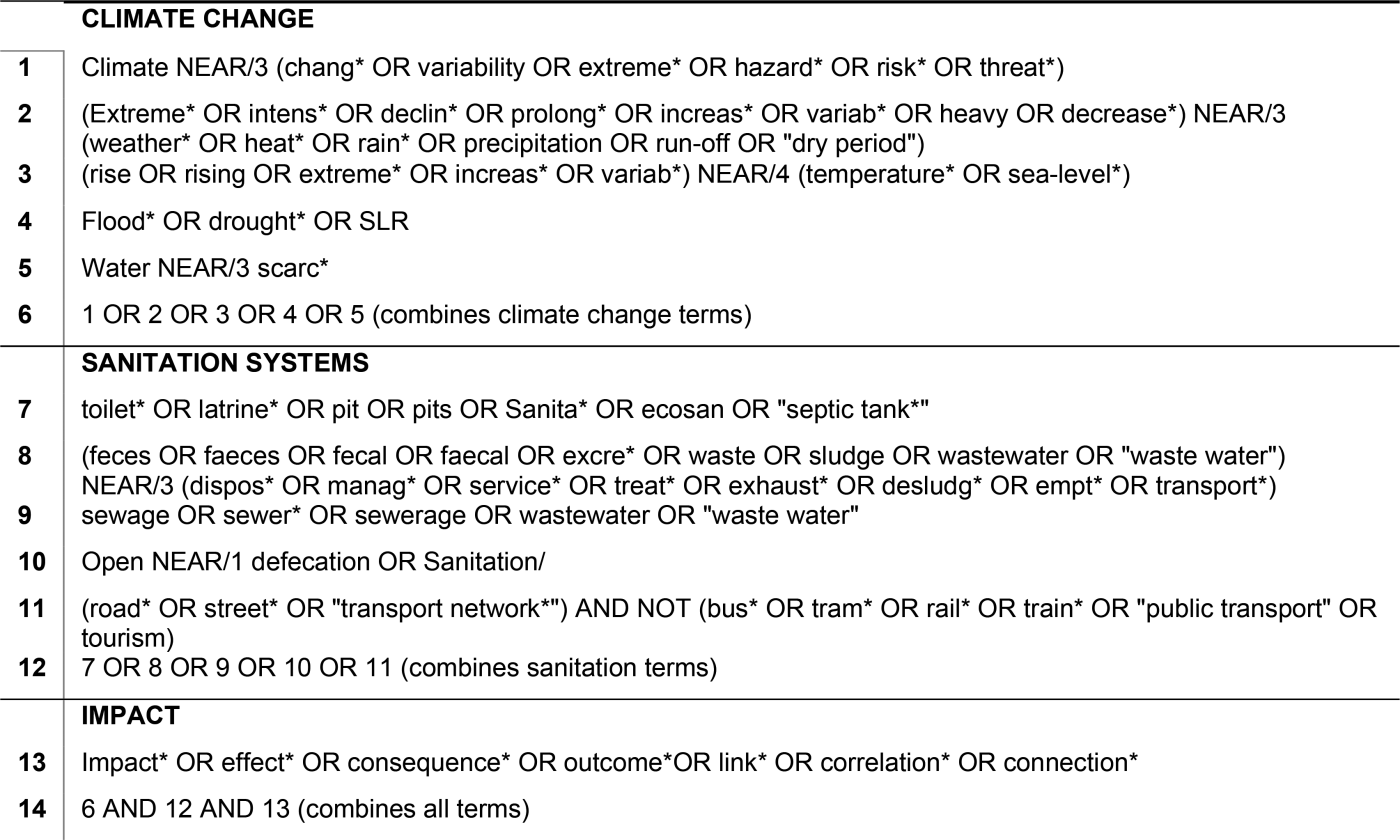
Electronic Database Search Strategy^a^

a This table presents the search strategy used to search the Web of Science database. The proximity operators have been adapted in compliance with the conventions of the respective database.

Search terms used for the gray literature search (Table S2) were tailored to the specific requirements and search capabilities of the included websites and databases. The results were supplemented by hand-searching the reference lists of selected studies and recent reviews. Experts in the field recommended additional literature that may have been missing from the results.

The systematic review of the literature involved the following stages:

Stage I: search of electronic databases and gray literature; results imported into reference management software (Endnote X9) and subsequently into the Rayyan QCRI web tool; removal of duplicates.
Stage II: screening of all database-retrieved titles by one author (L.H.-S.), with a second author (Z.Z.) independently screening 50% of the titles for quality control; discussion and resolving of disagreements with a third author (B.E.); abstract and subsequently full-text screening (including gray literature) conducted independently by two authors (L.H.-S. and Z.Z.) using the inclusion and exclusion criteria; discussion and resolving of disagreements with a third author (B.E.); selection of papers; hand-searching of references of excluded review papers and selected studies.
Stage III: final paper selection; data extraction by one author (L.H.-S.) with a second author (Z.Z.) assessing the accuracy of the extracted data for a subsample of 10% of the studies

L.H.-S. extracted and tabulated data from the selected studies by CC effect (or hazard) studied, the urban sanitation system (or component/process) covered, the method to study the impacts, and the quality of evidence (Tables S5–S7). Finally, we analyzed and mapped the selected papers according to the sanitation failure mode^26^ and the sanitation climate effect and hazard categories adapted from WHO (2019).^25^

### Failure Mode Analysis

The authors draw on a systematical analysis of urban sanitation failure modes (FM)^26^ to classify five urban sanitation FMs that result in human excreta being not safely managed and potentially causing public and environmental health risks: FM1, fecal sludge (FS) not contained and not emptied; FM2, FS and/or supernatant (SN) not delivered to treatment; FM3, FS and/or SN not treated; FM4, wastewater (WW) not delivered to treatment; FM5, WW not treated.^26^

For each study, we assessed the evidence that specific CC impacts acting on specific systems or components of systems increase or reduce the probability of each failure mode occurring.

### Quality of the Evidence Assessment

To evaluate the quality of the evidence, we used two appraisal categories: (i) relevance and generalizability of the presented evidence and (ii) general quality of reporting. We based the scoring of the first criteria on the following three subcategories:

Levels of evidence. On the basis of the study design, results were classified as empirical evidence, modeled or reported evidence, and expert consultation and each classification was ranked. Empirical evidence was given the highest score, followed by modeled and reported evidence (same scoring). Results of expert consultation received the lowest scoring. This ranking broadly follows the convention used in medical research^33,34^ and reflects the stronger representation of modeled evidence in engineering.
Scale and generalizability of reported impacts. We scored the scale and generalizability of reported impacts in three categories (in descending order for scoring): studies with global scale or case-independent approach, context-specific studies (in terms of climate impacts and sanitation systems) that are transferable to similar contexts, and very context-specific studies with limited generalizability.
Temporal scale. Studies describing impacts based on (likely) long-term climate trends or multiple occurrences of extreme events were scored of higher validity than studies presenting evidence based on observations during a single extreme event.

We adapted the quality appraisal framework developed by Venkataramanan et al.^35^ to evaluate the quality of reporting of both qualitative and quantitative papers. We modified their framework to reflect the nature of the included studies and the scope of this review (Table S3). In total, we used 10 criteria to score the included studies, each with a maximum score of 1. We evaluated papers with an aggregated score of 75% or above as “strong”. One author (L.H.-S.) scored all documents, and a second author (Z.Z.) independently scored a sample of 10% for quality control.

Table S4 presents details for the relevance and quality scoring of the included studies.

## Results and Discussion

### Screening and Selection

**Figure 1:**
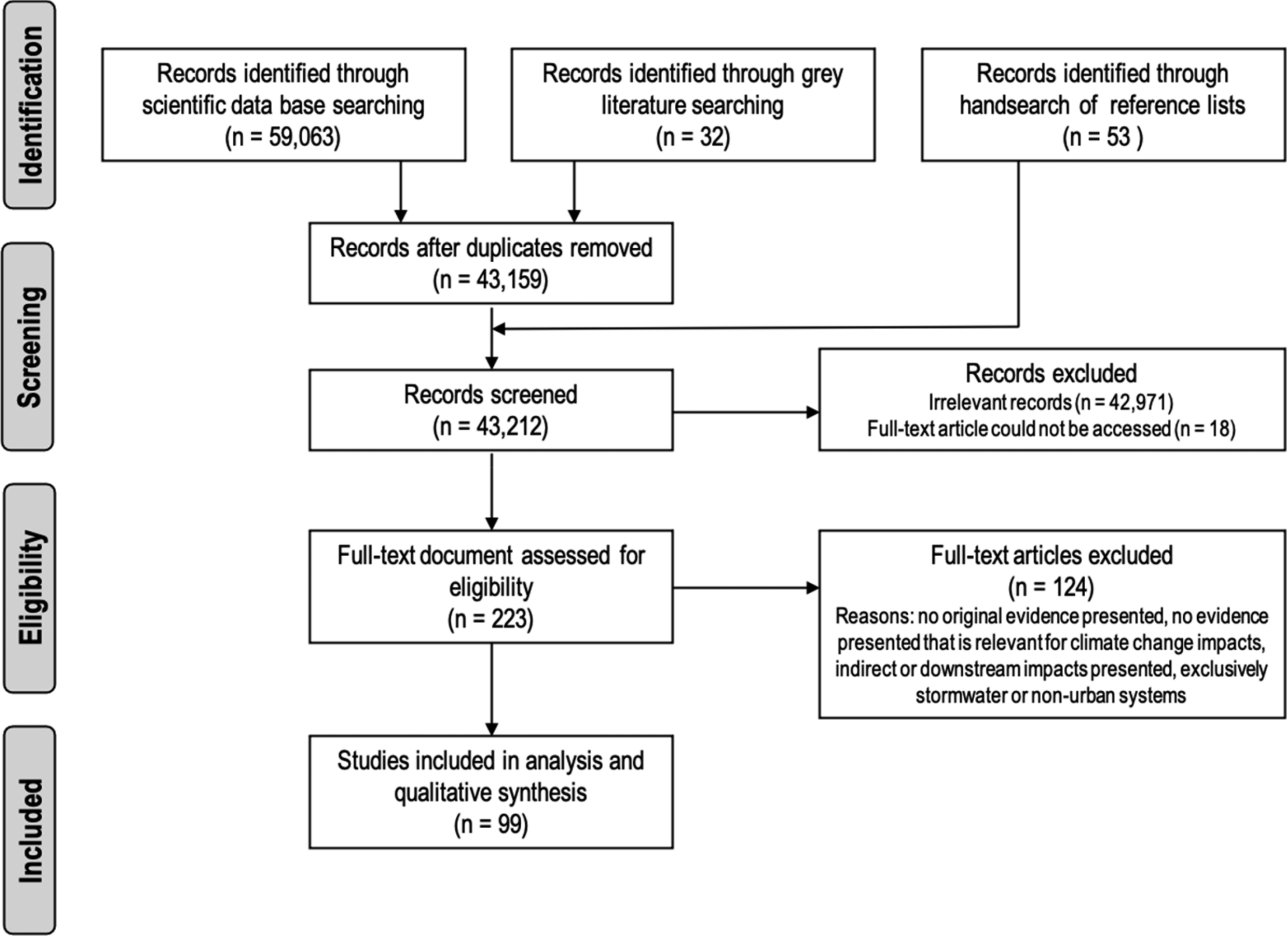
Flow diagram summarizing the screening and selection process.

The systematic search of databases retrieved 59 063 articles. Eighty-five records were identified through gray literature search (*n* = 32) and hand-searching reference lists of included articles and excluded review papers (*n* = 53). Expert consultation yielded no additional studies. A total of 15 936 duplicates were removed. Most of the remaining articles (42 971) did not relate to urban sanitation, CC impacts on sanitation systems, or analyzed indirect impacts of CC on sanitation (e.g., spread of diseases) or downstream effects. Eighteen papers identified through title and abstract screening could not be accessed for full-text review. A further 124 papers were excluded after content review. The main reasons for exclusion are listed in Figure 1.

### Characteristics of the Literature

#### Knowledge Clusters

We found that the evidence for impacts of CC on urban sanitation systems is contained in three separate clusters of work. First, there are sanitation studies coming primarily from the engineering literature and tending to focus on well-established technologies (mostly sewered systems and studies from HICs). The second cluster of studies are also from the sanitation sector but come from literature more closely embedded in international development, often interdisciplinary, but rarely based in the pure engineering literature and tend to focus on public health more broadly. It is impossible to differentiate these along purely technological lines because the first cluster also includes some nonsewered systems (e.g., septic systems in HIC contexts). Thus, we describe the first group as “primarily engineering” studies (*n* = 50), and the second as “sanitation and development” (*n* = 9). Finally, there are studies from the (road-based) transport sector (*n* = 40) (Table 2).

**Table 2:** Characteristics of the Included Literature

characteristics (*n* = 99)	no. of documents
Literature Type	
journal-published study	69
conference paper	21
gray literature	9
Type of Evidence	
empirical	25
modeled	47
reported	26
expert consultation	1
Knowledge Cluster	
sanitation sector studies	59
primarily engineering	50
sanitation and development	9
transport sector studies (road-based transport)	40
Sanitation System Category	
sewered sanitation	46
nonsewered sanitation (including road-based transport systems)	48
mixed (nonsewered and sewered sanitation)	5
Study Country Classification by Income^a^^,^^b^	
low-income economies	1
lower middle-income economies	13
upper middle-income economies	7
high-income economies	80
global	1

a According to the World Bank country and lending groups classification for the 2022 fiscal year (ref (36)).

b The sum of studies classified by income country classification is greater than 99 because some studies covered multiple countries.

#### Sanitation System Categories

Of the 59 sanitation sector studies, over three-quarters (*n* = 46) reported CC impacts on sewered systems. Half of those studies (*n* = 23) presented evidence for the impacts of CC on wastewater conveyance, 15 studies presented evidence on wastewater treatment, and eight studies covered CC impacts affecting aspects of both treatment and conveyance (wastewater management). All sewer studies relate to conventional (combined or separate) sewerage. We found no study presenting evidence for CC impacts on modified sewer systems.

We found evidence of CC impacts relevant for nonsewered sanitation systems in studies from the transport sector (*n* = 40) and the sanitation sector knowledge cluster (*n* = 8). Four of the latter reported on impacts on household latrines, and four reported on septic tank systems^37−40^ presenting data from a HIC context.

Five studies reported on mixed sanitation systems. Four of these^41−44^ referred to mixed systems in specific communities, and one study presented a global assessment of CC effects on various sanitation technologies.^11^

None of the included studies explicitly explored the impacts of CC on the transport of FS. The focus of the road-based transport systems was divided between studies investigating the impacts of CC on infrastructure (*n* = 23) and the performance of the transport network (*n* = 16). One study covered both aspects. Details of all included studies are provided in Tables S5–S7.

#### Type of Evidence

**Figure 2:**
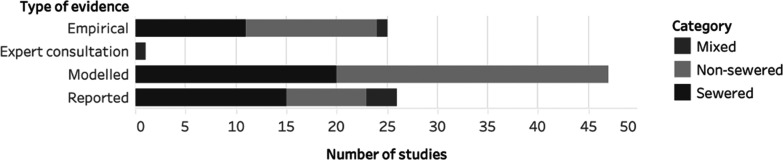
Distribution of study populations and evidence type among included studies. The modeling approach varied in the different studies and included sewer and transport flow modeling, downscaled global circulation models, stochastic modeling or projections based on δ-change methods, etc. More details on the applied modeling approaches are provided in Tables S5–S7.

Around a quarter (*n* = 25) of the studies presented empirical evidence. Forty-seven studies presented modeled data, and 26 presented reported evidence (i.e., results from surveys, interviews or data extracted from operational records). One study showed the results of a structured expert consultation. The highest proportion of modeled data was observed among the wastewater conveyance studies, with 18 out of 23 studies using a model-based approach. Modeling was also common in the transport sector studies (24 out of 40 studies) (Figure 2). Within the “sanitation and development” knowledge cluster, only one study presented empirical evidence.

#### Climate Change Effects

The most common CC effect was changing precipitation intensity or frequency, including pluvial flooding (*n* = 41). Ten of those studies also included another climate effect such as a change in temperature (*n* = 6), sea-level rise (SLR) (*n* = 2), rising groundwater levels (*n* = 1), or extreme weather (*n* = 1). Twenty-six studies presented evidence on extreme weather (such as storms, heat waves, or extreme rainfall events). Eight studies addressed the impact of SLR, and two studies presented results for both extreme weather (including storm surge) and SLR. Seven studies described the impacts of temperature changes and variations, and four investigated drought impacts. Five studies related to various climate effects, and another five investigated the impacts and interrelation of a specific combination of climate effects.

#### Coverage

We found an over-representation from studies presenting data from HICs: over half of the studies (*n* = 55) were from just three countries: the United States (*n* = 39), Canada (*n* = 9), and the United Kingdom (*n* = 8). Eighty-six of the studies assessed impacts on systems in upper-middle and high-income countries. One study had a global focus, and the remaining 12 studies covered evidence from LMICs (or multiple countries in this category). A graphic illustration of the regional distribution of the studies is shown in Figure S1.

### Relevance of Evidence and Quality of Reporting

According to our scoring criteria, 86 of the included studies reached at least 50% aggregated score (Table S5). Forty scored 75% or higher of the total maximum score (strong studies). Most of the “strong” studies were either in the transport sector or “primarily engineering” sanitation sector cluster and published in journal articles; however, there was no strong trend in terms of quality of evidence along the sanitation chain. Almost half (12 out of 26) of the studies presenting evidence on extreme weather events were published in conference papers, with often lower reporting quality scoring when compared to published journal papers.

### Impacts along the Sanitation Service Chain

This section describes the impacts of CC on sanitation systems as presented in the included studies along the sanitation service chain.

#### Impacts on the Use of Sanitation Systems and Containment

Most studies reporting CC impacts on access to and use of toilets themselves relate to nonsewered sanitation systems and the impact of flooding and extreme rainfall events. Four studies report structural damage to pits or the superstructure and overflowing of toilets.^11,45−47^ Few specify whether the damage or contamination occurred due to surface or groundwater flooding. None of these studies specified the extent to which inadequate maintenance contributed to the extent of the failure or collapse. Rising groundwater tables (due to increased rainfall or SLR) were connected to increased pollutant mobility within the soil-based treatment area of septic tank systems, and one study linked this to increased nitrogen contamination of groundwater and surface water bodies.^39^ In informal settlements in the Philippines, flooding was also responsible for the malfunctioning of water-based toilets due to electricity failure resulting in a lack of water supply.^43^

Inundation and inaccessibility of sanitation systems led to (temporary) changes in sanitation behaviors. Coping mechanisms included switching to a different type of sanitation, which included unsafe sanitation behaviors.^41,43,45−48^ In Bangladesh, people reverted to open defecation,^41,48^ and in informal areas in Antananarivo, the use of “flying toilets” (defecating into a plastic bag) increased.^47^ Drought also triggered coping mechanisms. In Botswana, people stopped using flush toilets connected to a sewer system due to water restrictions and shortages during drought events. Common alternatives were pit latrines. Leachate from those pit latrines was suggested as a likely source of groundwater pollution.^44^ However, the study could not completely rule out alternative sources for the detected NO_3_ and pathogens contamination.

Other negative impacts of extended dry periods on containment systems included structural damage to toilets caused by erosion in low-moisture soils^11^ and decreasing levels of hydroelectric productivity resulting in failure of groundwater pumps that provided water for pour-flush toilets in low-income settlements in Accra, Ghana.^42^ However, due to the complexity of the underlying reasons for electricity failures in Ghana, the evidence could not unambiguously be linked to reduced hydropower production. As a positive impact of declining rainfall, declining groundwater levels might reduce groundwater pollution risk from onsite containment systems,^11^ albeit none of the identified studies presented empirical evidence for this link.

#### Impacts on Emptying and Conveyance

Only one study suggests a potential direct impact of climate effects on toilet emptying practices. On the basis of experiences during the rainy season in Dar-es-Salaam presented by Chaggu et al.,^49^ the Vision 2030 research proposes the “risks from flooding may be exacerbated by owners using floodwater to flush out the latrine pits” (ref (11), p 18). While this statement appears to be a valid assumption, the original study^49^ does not refer to CC impacts on this or other sanitation practices.

CC impacts on road-based transport systems can be divided into long- and short-term impacts of the integrity of road pavement (*n* = 20),^200−68^ or other structural elements of the network (e.g., bridges) (*n* = 3),^69−71^ and disruption of transport network performance or capacity, such as inaccessible roads, increased congestion, and travel time (*n* = 16).^72−88^ Alteration of transport network performance was commonly measured with indicators such as changes to network accessibility, the ratio of accessible network length, vehicle hours traveled, vehicle miles traveled, trips completed, and loss in connectivity. Most studies reporting on physical infrastructure implications associated with CC effects presented evidence for temperature changes or flooding impacts for road pavements. Disruption of transport network performance was mainly attributed to intense rainfall or flooding caused by extreme weather or SLR. However, several studies qualified the predicted impact of CC on the pavement infrastructure as relatively small compared to other factors such as seasonal weather variability or increase in future traffic.^57−59^

The bulk of sewerage studies examine the relationship between changes in the frequency and intensity of precipitation events and the efficacy of the sewer conveyance system in terms of duration, frequency or spill volumes of combined sewer overflows (CSOs),^11,89−105^ or increased risk of urban flooding due to backflow of sewage, overflowing inspection chambers, or flooding of basements.^92−94,98,100,101,106−108^ Some studies also linked the increased volume or frequency of CSOs to higher pollutant concentrations in receiving water bodies.^92,97,99,102^ The scale of these impacts could be plausibly linked to the preflood condition of the sewer system, which in turn is linked to the level of ongoing maintenance.

Reported impacts of flooding or high-intensity rainfall events on sewer infrastructure were damages to sewer pumps and mains,^11,109−111^ including increased risk of pipe failure due to changed soil moisture and associated subsidence,^101^ and sewer blockages after flooding events caused by sand, debris, or solid waste entering the system.^41,112^ For storm events, it was reported that extreme winds caused the uprooting of trees, which damaged sewer pipes, as did the replacement of electricity poles and the deployment of heavy equipment during the cleanup following extreme weather events.^113^

In this context, it is important to recognize that extreme weather events have immediate and delayed impacts on sanitation systems. Most studies focused on the immediate impacts during and after extreme rainfall or storm events, but—as the examples above illustrate—some scholars also demonstrate delayed or long-term implications of extreme weather events.

Sewer system service disruptions caused by flooding and storm events were caused by sewer pump failures resulting from electricity outages.^109^ Several studies presented evidence of the reduced capacity of sewer systems caused by increased inflow and infiltration due to intense rainfall/flooding^101,112,114,115^ or associated with SLR.^115−118^

However, various studies showed that the effects of urbanization might have similar impacts on sewer systems as changes in precipitation patterns and will exacerbate the impacts of CC.^97,98,105,108,114^ Inadequate maintenance leading to poor condition of many sewer systems reduces their resilience during extreme weather events and aggravates the damage caused by those events.^110,119^ The potential increase of inflow and infiltration into separate sewer systems from SLR will depend on the system’s technical status.^116^

SLR was also associated with higher groundwater tables and thus risk of pollution from leaking pipes^117^ and corrosion of pipes through saltwater infiltration.^101^ In coastal areas, the combination of SLR, storm surge, extreme tides, and rainfall events can compromise combined sewer discharge facilities if the hydraulic head of wet weather flow is insufficient to force water through backflow prevention devices leading to sewer backup and potential flooding at low points of the sewer network.^11,101,120^

During drought events, reduced flow rates and higher concentrations of wastewater associated with water conservation were found to cause buildup of solids and subsequently blockages in sewer and discharge pipes^11,121−123^ and contribute to increased sewer corrosion and odors due to the generation of acids and odorous gases.^101,122^ Due to changing moisture content, soil movements increased the risk of pipe and joint breakages, particularly in soils with high clay content.^11^ All of these effects would be exacerbated in poorly maintained systems. Some studies also reported the positive impacts of drier weather on the efficacy of sewer conveyance systems, such as the reduced risk of overflowing inspection chambers^103,107^ and decreased CSO spill frequency and volume.^91,95,103^ Potentially limiting the benefits of the latter, CSO spills within or after periods of drier weather were found to cause higher pollutant concentrations due to lower water levels and thus reduced dilution in receiving water bodies.^102^ By contrast, lower groundwater levels are thought to reduce the risk for groundwater contamination from pathogens.^11^

An important observation for cities with complex sanitation systems comprising sewer networks and FSM systems was that damage to^110,111,113,119^ or overload of^75^ sewer systems could disrupt road infrastructure or road-based transport network performance. Various authors described damages to sewer pipes that led to soil destabilization and ultimately partial road collapse (e.g., the occurrence of sinkholes).^110,111,113,119^ However, none of the studies established the logical continuation of this impact chain; in cities (partially) relying on nonsewered sanitation, this could ultimately lead to a breakdown of fecal sludge collection. This lack of coordinated consideration of climate impacts mirrors the lack of integrated management and operation of sanitation systems reported by Peal et al.^124^ and others.

#### Impacts on Wastewater and FS Treatment

Almost all the studies that report CC impacts on treatment systems relate to wastewater treatment facilities. Four studies presented evidence on the impacts of various climatic factors on septic tank systems.^37−40^ Noticeably, there is inconsistency in nomenclature to describe these systems. In older studies^40^ and studies from the “sanitation and development” cluster (e.g., refs (11 and 47)), “septic tank” or “septic tank systems” is used. In contrast, more recent studies^37−39^ use the term “onsite wastewater treatment systems” to describe systems consisting of “a septic tank, drainfield and the native soils” (ref (39), p 1874). Since those systems also act as containment, we presented part of the evidence in the section above. Moderate increases in soil temperature were associated with increased contaminant removal capacity in septic tank systems.^37,38,40^

Almost all studies referring to potential impacts of CC on FS treatment present evidence for impacts on soil-based treatment in septic tank systems in high-income low-density contexts.^37−40^ In dense urban settings, stand-alone septic tanks are rarely a suitable sanitation solution at scale because soil-based treatment of the liquid fraction is not viable due to space constraints and limits of the soil treatment capacity.^125^ Research has shown that in cities, where large parts of the population rely on septic tanks, operation and maintenance, including regularly emptying and further sludge treatment, is often inadequate.^126^ Septic tank systems are frequently poorly constructed, with the liquid supernatant usually ending up in the drainage network,^31^ potentially giving rise to blockages and further flooding.^26,126,127^

Wastewater treatment plants (WWTPs) are frequently located in low-lying zones and are vulnerable to flooding during intense rainfall and extreme weather events. In coastal cities, WWTPs are also exposed to flood risk due to SLR and storm surges during extreme weather events (e.g., hurricanes and cyclones). Various studies presented evidence for flood waters causing damage to WWTP infrastructure and equipment.^11,89,109−112,128^ Inundation with seawater was found to be more damaging to equipment^112,117^ than freshwater inundation. Corrosion of treatment equipment was also reported due to drought, causing more concentrated and corrosive influent to WWTPs.^122^ Water scarcity has previously been proposed as a plausible constraint on the implementation or sustained operation of sewerage,^129^ but there was limited empirical evidence to support this. A study assessing the impacts of low flow due to droughts and related water conservation measures concluded that excess depositions and siltation from up to 20% reduced flow rates was negligible for most parts of the WWTP and might only be of concern in velocity-controlled grit chambers.^130^ Experience from earthquake-induced land subsidence in Japan suggested that SLR-induced rising groundwater levels might generate buoyant forces in areas not designed for high groundwaters and thus damage buried infrastructure such as pipes.^117^

Evidence for service disruptions of wastewater treatment plants mainly referred to (temporary) system failures due to flooding of facilities^92,110,111,113,128^ or overloading of sewers resulting in bypassing treatment.^110,128^ The importance of interdependent urban infrastructure was demonstrated by studies reporting that during flood events road interruptions and closures led to disruption in staff and supply access to WWTPs^109−112,128^ and electricity outages caused failures of pumps and pond aeration;^112,128^ SLR in combination with high tides was predicted to limit the ability to discharge treated wastewater into water bodies by gravity and cause backflow into the system.^120,122^

CC-related impacts on the efficacy of treatment systems included extreme rainfall events during which increased pollutant loads of the influent can exceed the biological treatment capacities of the WWTP^121,131,132^ and reduce retention times^132^ leading to reduced nutrient removal. Lack of maintenance may result in separate sewer systems experiencing increased inflow and infiltration during rainfall events and de facto behaving like combined sewer systems. A study from Zimbabwe demonstrated that the treatment efficacy of WWTP connected to such a structurally unsound separate sewer system declined during intense rainfall events as the inflow rates and loads exceeded the design parameters of the treatment plant.^132^ SLR was found to cause higher inflow and infiltration, stretching the design capacity of WWTPs.^115,118^ However, high-intensity rainfall events were also associated with more diluted inflow into WWTPs, positively affecting effluent quality.^114,131^

Due to more concentrated wastewater inflow, declining rainfall and prolonged dry periods were associated with reduced discharge quality.^122,130,133,134^ For seawater-induced flooding events, inundation of WWTP with saltwater was linked to a negative impact on biological treatment processes.^112^

Temperature variations can positively or negatively impact the efficacy of treatment processes. Several studies linked moderate temperature increases to improved removal efficiencies in WWTPs^133,135^ and FS treatment systems.^38,40^ However, more extreme temperature shocks were found to reduce biological treatment efficiency.^136^ Two studies investigated the effects of winter temperature variations leading to snowmelt and thus a sharp decrease of wastewater influent temperature, which reduced treatment efficiency.^137,138^ Overloading or bypassing treatment plants was found to contaminate receiving water bodies.^92,121,128,139^ In terms of environmental risk, treatment efficacy is interlinked with the dilution capacity of receiving water bodies, which is expected to decrease for drier weather.^11^

Overall, the literature provides evidence of multiple impacts of CC on sewer conveyance and wastewater treatment. The evidence for impacts on nonsewered sanitation is more limited, with few studies providing examples of the failure of pits and tanks and users reverting to unsafe sanitation practices primarily during flood events. While never making this explicit, the studies which look at sewerage, road networks, and treatment plants often imply the interconnected nature of the urban system and the potential for prolonged multiple failures in cities relying on both sewered and nonsewered sanitation under extreme weather conditions.

### Failure Mode Analysis

To explore the literature landscape in more detail, we linked evidence about CC impacts to existing knowledge of the modes in which urban sanitation systems fail to provide safe sanitation.^26^

Table 3 shows that available evidence on how CC will likely increase or reduce the probability of typical modes of sanitation failure concentrates on the management and treatment of wastewater in sewered sanitation systems (failure modes 4 and 5) and climate impacts on road-based transport systems. When excluding the 40 studies from the transport sector cluster, only 11 studies presented evidence relevant to the FSM failure modes (FM 1–3). Almost all of those studies discussed the impacts of CC on onsite sanitation containment, with only three studies referring to damaged road networks. However, no studies explicitly investigated how climate effects impact FS conveyance services. As mentioned earlier, only one study presented evidence for the potential impacts of CC on FS emptying (part of FM2). There is scant evidence for the impacts of CC on fecal sludge treatment (FM3). In general, the tabulation reveals a clear dominance of evidence referring to impacts of CC on sanitation infrastructure, whereas there are few studies that present evidence for the implications of CC on urban sanitation service provision and management.

**Table 3:**
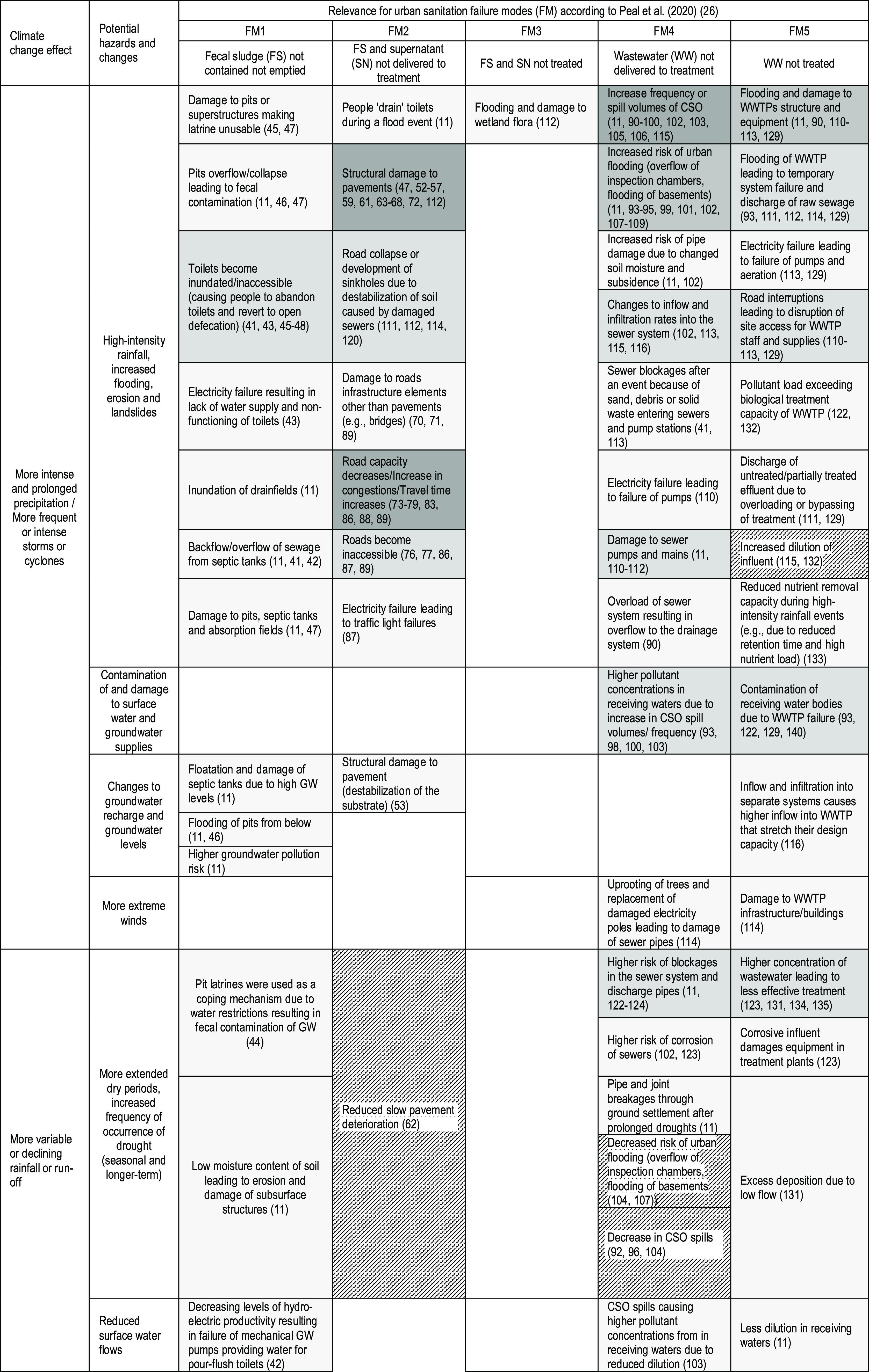

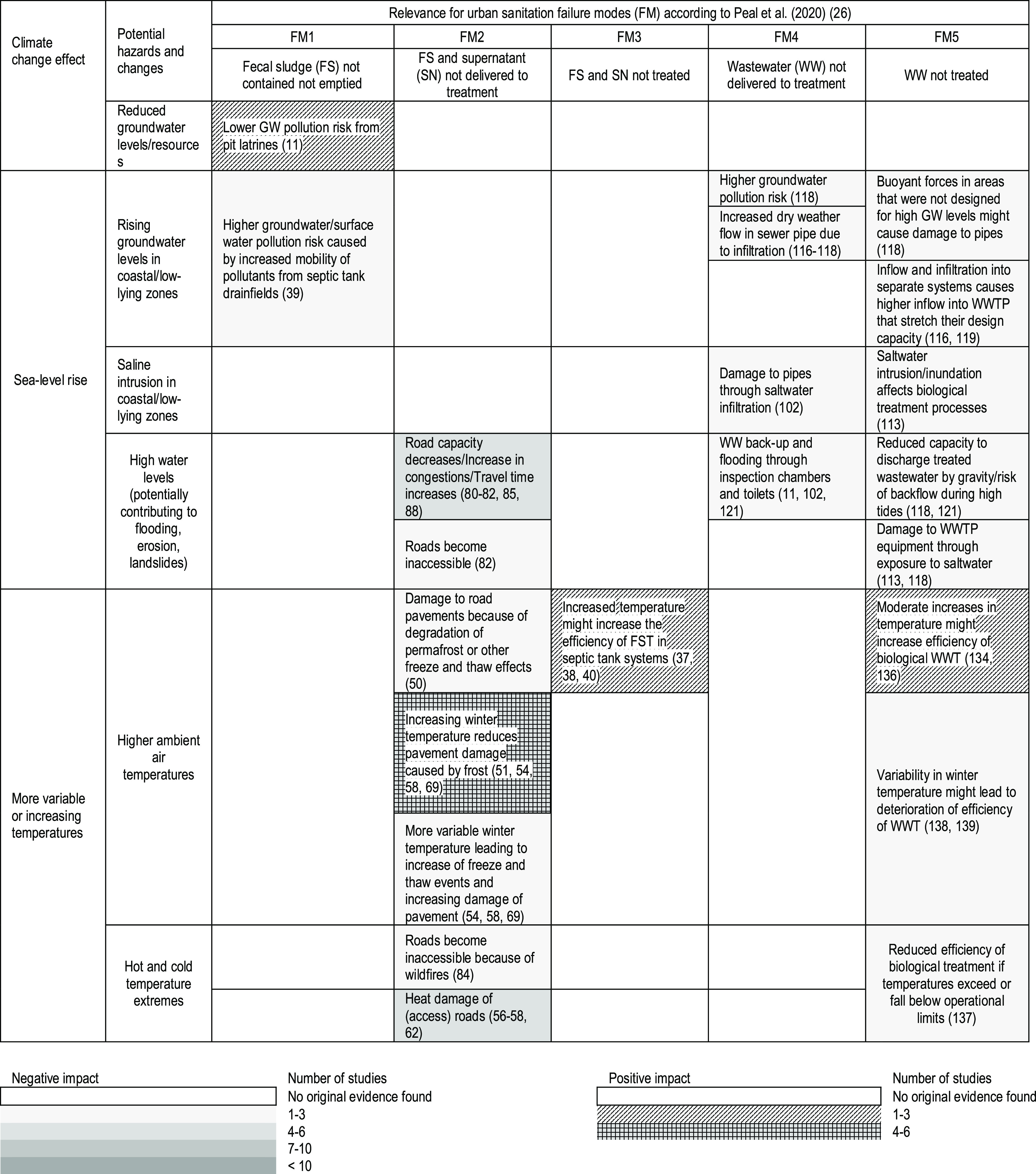
Mapping of Evidence of Climate Change Impacts on Urban Sanitation System along the Sanitation Failure Mode Classification (*n* = 99)

In Table S8, we present a version of the failure mode matrix including only evidence from “strong” studies (*n* = 40). Table 4 shows a comparison of the number of individual studies and impact categories for each failure mode category before and after quality and relevance scoring. Before scoring, over 40% (33 out of 76) of the impact categories (cells of the failure mode table) rely on single-source evidence. After quality scoring, this proportion increases to over 50% (29 out of 55) of the studies. Eleven out of those 29 impact categories relying on single-source evidence are based solely on the Vision 2030 research,^11^ which derived its original evidence solely from expert judgment.

**Table 4:** Comparison of the Number of Studies and Impact Categories per Failure Mode Category before and after Quality Scoring

	FM1	FM2	FM3	FM4	FM5	total
All Studies Included (see Table 3) (*n* = 99)						
no. of individual studies	10	44	4	35	26	99
no. of impact categories	15	16	2	20	23	76
no. of impact categories relying on evidence from a single source	9	7	1	7	9	33
Only Studies Scoring 75% or Higher in Aggregated Relevance of Evidence and Quality of Reporting Score (see Table S5) (*n* = 40)						
no. of individual studies	6	27	3	21	14	40
no. of impact categories	15	12	1	14	13	55
no. of impact categories relying on evidence from a single source	11	4	0	5	9	29

The tabulation also highlights the uneven distribution of studies between the failure mode categories. After scoring, the total number of impact categories for which evidence was available in at least one of the included studies is reduced from 76 to 55. We observed the highest postquality scoring reduction of evidence-based impact categories (from 23 to 13) and relevant individual studies (from 26 to 14) in the FM5 category. Noticeably, there was no reduction of impact categories under the FM1 category, but postscoring 11 out of 15 impact categories in this cluster rely on single source-based evidence.

### Drivers of Poor-Quality Evidence

We found that the evidence for CC impacts on urban sanitation systems is weak. Many proposed impacts are demonstrated from a single source based only on expert judgment.^11^ Despite screening over 43 000 search results and including keywords beyond explicit reference to CC, we only found 59 studies that explicitly presented evidence for potential and actual direct impacts of a changing climate on the management of human excreta in urban areas. The available evidence concentrates on sewerage and wastewater treatment systems and experiences from high- and upper-middle income countries. The majority of papers used models to predict CC impacts on the sanitation system. As models are always a simplified version of reality, such a heavy reliance on model-based studies might limit understanding of more complex interactions of CC effects and their impacts on sanitation systems. Unexpected CC impacts—notably cascading and interlinked impacts—will not be represented.^121^

A substantial proportion of the included studies (40 out of 99) presented evidence for CC impacts on road-based transport systems. Our review found that, so far, the evidence from the transport sector is not adequately accessed, transferred, and expanded from and into the sanitation sector.

### Overarching Themes

The review identified several overarching themes which we discuss below, including a lack of consideration of urban FSM, lack of recognition of interdependencies between infrastructure and service systems, complexity of CC effects, interdependence with other urban sectors, and limitations of autonomous household adaptation.

#### Lack of Consideration of Urban Fecal Sludge Management

The post-2015 update of the WHO’s Vision 2030 acknowledges the potential vulnerability of FSM to CC and the disruption that flooded roads might cause for emptying vehicles^4^ but does not provide original evidence to support this concern. We found only sparse evidence on the impacts of CC on urban FSM in the reviewed studies. Only one study^11^ mentioned the potential impacts of CC on FS emptying practices. While sufficient evidence from the transport sector generally describes how road-based transport systems could be impacted by CC-induced infrastructure damage or network performance and capacity disruptions, no study explicitly identifies the implications for FS emptying and transport services. There are also no studies exploring FSM service chains by linking fecal sludge emptying and transport disruptions to impacts on FS treatment systems.

#### Lack of Recognition of Interdependencies of Urban Sanitation Infrastructure and Service Systems

Our review found that the evidence for the impacts of CC on urban sanitation systems is contained in separate clusters of work that are poorly connected. Most studies on urban sewerage presented evidence from HIC contexts where homogeneous urban sanitation systems dominate.^29^ Most studies presenting evidence from cities in LMICs with complex and fragmented sanitation systems used relatively homogeneous areas (in most cases low-income settlements) as case studies to describe the impacts on their respective sanitation systems (mainly nonsewered sanitation). None of the reviewed studies investigated the impacts of CC on a citywide complex sanitation system featuring a mixture of centralized sewerage and nonsewered decentralized sanitation systems. This conscious or unconscious “insulation” of sanitation infrastructure and services systems does not reflect the reality of sanitation systems in many cities globally;^29^ there is limited acknowledgment of the interconnectivity of different sanitation infrastructure and service systems within one city.^140^ This suggests a lack of systems thinking in the sanitation sector and a prevailing focus on technologies rather than service approaches.^141^

#### Complexity of Climate Change Effects

CC impacts on urban sanitation systems are complex, and combinations of climate effects need to be considered. While most studies looked at a single CC impact, a few studies demonstrated the importance of acknowledging the complex interaction of CC impacts. Langeveld et al.^121^ showed that the impacts of an extreme rainfall event were exacerbated by a preceding prolonged dry period. A combination of extended dry periods and more intense rainfalls have been predicted for various geographic regions.^142^

Further variations in the predicted changes and extremes might be more critical than average changes. Multiple studies demonstrated that, despite minor changes in total annual rainfall volumes, the increase in shorter and more intense rainfall events would substantially impact the performance of current sanitation systems, which require major investments to adapt to these changes.^91,104,105^ Analogously, variations in average temperature and long-term temperature changes have moderate effects on system performances such as wastewater treatment processes or the condition of pavement structures. By contrast, in colder climates, rapid and large changes in winter temperature have substantial impacts on treatment processes^137,138^ and road pavement stability.^53,57,68^

#### Interdependencies with Other Urban Sectors

Urban sanitation systems have interdependencies with other urban sectors and services.^140^ Our review acknowledged the importance of road-based transport systems as intrinsic components of FSM services and revealed evidence for the knock-on effects of electricity outages.^42,43,109,112,128^ Particularly in areas where increases in the frequency and intensity of heavy rainfall events are predicted, efficient urban drainage and solid waste management systems are crucial for the functioning of urban sanitation systems.^140,143^ While there is a growing body of literature in the urban disaster risk sphere exploring cascading effects of disaster and interdependencies of critical infrastructure (e.g., ref (144)), the interdependencies of poorly functioning sanitation systems with other urban infrastructure and services in the context of CC are not adequately researched. Neither is there evidence to help policymakers prioritize management strategies to reduce these cascading interconnections.

#### Limitations of Autonomous Household Adaptation

On the basis of the rationale that globally (and particularly in LMICs), sanitation relies heavily on household management and that even poor households can adapt (onsite) toilet designs and thus cope with climatic impacts threatening the functioning of their sanitation systems, the Vision 2030 research^11,12^ concludes that the resilience of sanitation systems is more driven by technology than management. Evidence included in this review contradicts this hypothesis. Postflooding, people reverted to open defecation^41^ or flying toilets.^47^ In Botswana, drought-induced sanitation behavior change potentially led to a loss of efficacy of sanitation systems to protect environmental and public health.^44^ Another study found no long-term adaptation of water supply or sanitation systems: “People just try to pass the days of flood anyhow and do the same every year; they do not do anything that will support them during the next flood” (ref (48), p 311). In low-income areas in Manila, the Philippines, Purwar et al.^43^ suggested that increased frequency of floods will reduce the priority of households to adapt to flood.

### Limitations

We excluded downstream effects from the scope of this review. However, this limited the inclusion of papers showing cascading impacts of CC, such as the combined effects of increasing CSO discharge, warmer water temperatures, and lower water levels in receiving water bodies resulting in an increased risk of waterborne disease.^145^ We limited our search to publications in English only, which might have under-represented research from non-Anglophone countries. A considerable body of literature reports on the effects of weather, mainly rainfall, on road-based transport systems. A high-level review of those papers indicates that they reinforce the presented results on the likely impacts of CC; however, we excluded studies referring to the impact of “normal” daily and seasonal weather variations (e.g., impacts of rain on traffic flow). There is a risk of bias toward studies explicitly stating negative impacts of a particular climate trend while the positive outcomes of the reverse trend are not reported.

### Implications and Perspectives

This is the first systematic review to assess the evidence of CC impacts on all types of urban sanitation systems, considering the existing knowledge on urban sanitation failures, and integrating the available evidence for CC impacts on urban road infrastructure and network performance. In the road-based transport knowledge cluster, we found a substantial body of literature that could inform adaptation and resilience planning for urban FS transport and decentralized sanitation systems. However, a lack of intersectoral thinking means that sanitation scholars and practitioners currently overlook this knowledge cluster.

Our review has highlighted that the research on urban sanitation is skewed toward studies that assess the impacts of CC on centralized, highly engineered, high-cost sanitation options situated in high-income contexts. In addition, we found that most evidence for CC impacts on sanitation systems refers to infrastructure rather than operational components. While lack of attention (and funding) for operation and maintenance of sanitation and specifically FSM systems is widely acknowledged in the sanitation sector,^146^ the lack of evidence for the impacts of CC on the operational side of FSM remains startling. The latest Joint Monitoring Program data shows that globally nonsewered sanitation infrastructure (septic tank systems and pit latrines) in urban areas has been increasing at twice the rate of sewer connections (ref (2), p 54). Research has shown that non- or mismanagement of fecal sludge and supernatant (FM1–FM3) contributes substantially to unsafe urban sanitation management.^26,31,126^ The impacts of CC are likely to aggravate existing challenges further.^25^ One possible explanation for this FSM “blind spot” could be that nonsewered sanitation is still considered “household managed”.^11^ However, the lack of evidence for autonomous household adaptation capacity to the impacts of CC on sanitation systems suggests that a planned public service approach at city level is required to actively manage and adapt sewered and nonsewered sanitation systems. Particularly in fast-growing cities and towns in LMICs, this is essential since sewer-based sanitation services are not keeping pace with urbanization.^2,141^ In addition, an increasing number of urban dwellers are projected to live in areas affected by severe water stress where the expansion of water-based conveyance systems will be limited by competing pressures on limited water resources.^129^ Therefore, onsite containment and effective FSM services will be necessary for the foreseeable future.^141^

Lack of relevant data and evidence is limiting the ability of countries to successfully submit applications for funding for sanitation adaptation and resilience projects.^20^ In particular, the multilateral climate funds, including the Green Climate Fund, the Global Environment Facility, and the Adaptation Fund, are focused on additionality and require applications to provide clear evidence and metrics demonstrating how the proposed projects and programs contribute to climate goals as opposed to broader societal development.^147^ Incremental costs of “hard”, infrastructure components are easier to identify and appraise in terms of their additionality, which is reflected in a preference of “hard” over “soft” components, including operational adjustments in sanitation adaptation and resilience funding disbursements.^20,147,148^

We are concerned that the current focus of research related to the impacts of CC not only contradicts the sector’s future trends but will also influence the focus, quality, and robustness of sanitation future adaptation and resilience measures. Investments in infrastructure alone will not render a sanitation system “resilient” toward the impacts of CC.^149^ Lack of understanding and anticipation of the impacts of CC on complex sanitation systems in contexts that are already less well-resourced and have lower institutional adaptation capacities is likely to reinforce existing sanitation inequalities and vulnerabilities through climate adaptation projects and investments.

## Supporting Information Available

Inclusion and exclusion criteria for the systematic review, list of websites for and gray literature searches, and respective search strategy, appraisal framework for evaluating the quality of reporting and relevance of evidence for the included studies, results of relevance of evidence and quality of reporting evaluation for all included studies, details of included literature in the systematic review, failure mode matrix including only results from “strong” studies, and regional distribution of evidence (PDF)
