# Supplementary material for: Climate Change Impacts on Urban Sanitation: A Systematic Review and Failure Mode Analysis
Source: Environ Sci Technol. 2022 Apr 12;56(9):5306–21. doi: 10.1021/acs.est.1c07424 (PMC9069703; doi:10.1021/acs.est.1c07424)
Supplement: Supplementary file 1 — es1c07424_si_001.pdf [file es1c07424_si_001.pdf]

SUPPLEMENTAL INFORMANTION

Climate change impacts on urban sanitation: a systematic review and failure mode analysis

Leonie Hyde-Smith†, Zhe Zhan†, Katy Roelich†, Anna Mdee†, and Barbara Evans*,†

† University of Leeds, Woodhouse Lane, Leeds LS2 9JT, UK

*Corresponding author's email address: b.e.evans@leeds.ac.uk

The supplemental information is 43 pages and contains 8 tables and 1 figure.

Supplemental Information, Table S1. Inclusion and exclusion criteria for the systematic review

CATEGORY	Included	Excluded
Population	Systems or components of systems that are typically part of urban sanitation systems including infrastructure (e.g., sewers or pit latrines) or services (e.g., transport services for faecal sludge)	Systems or parts of systems which are exclusively or almost exclusively used in rural places (e.g., arboloos, studies that report on impacts on rural CLTS programmes)<br><br>Urban drainage systems that are exclusively used for stormwater
Category of evidence on outcomes	Empirical evidence, reported evidence, modelled data, expert consultation, expert opinions	Advocacy pieces, Review papers that do not add new evidence
Outcomes	Direct positive and negative climate-related impacts on urban sanitation systems that affect the delivery of safely managed sanitation.<br><br>*Negative impacts* include: damage on sanitation infrastructure (e.g., blockage or corrosion of pipes), disruption of services (e.g., inaccessible roads for emptying trucks, failure of sewer pumps and treatment processes due to electricity outages) and inhibited efficacy of the system (reduced efficacy of biological wastewater treatment processes, excessive leakage)<br><br>*Positive impacts* include: prolonged life or reduced maintenance requirements of infrastructure (e.g., slower filling up of pit latrines), improved service delivery (less disruption of emptying services), improvement in system performance (e.g., improved efficiency in treatment processes)	Positive or negative secondary impacts on human and environmental health and the economy (e.g., increased household or public expenditure on repairs or preventive maintenance, increase in specific waterborne diseases (e.g., cholera) after extreme weather events, increased business opportunities for emptying businesses, fish mortality or reduced biodiversity in receiving water bodies)<br><br>Positive or negative non-economic secondary impacts (e.g., loss of privacy through the collapse of latrine superstructures, increased absenteeism due to the damage of school toilets)
Study types and designs	Qualitative and quantitative studies that assess or report on specific climate change impacts on urban sanitation systems	Studies that do not specify climate change impacts.<br><br>Studies that exclusively refer to the impacts from a different study (e.g., to test adaptation measures). Studies that exclusively refer to non-climatic impacts on sanitation systems (e.g., urbanisation impacts)
Publication types	Published journal article and published conference proceedings, consultancy and agency reports (grey literature)	Editorials, editorial, short opinion pieces, letters, newspaper articles, books and book sections
Publication dates	No limit	
Publication language(s)	English	

Supplemental Information, Table S2. List of websites for and grey literature searches, and respective search strategy.

DATABASE OR WEBSITE	Search strategy
**SuSanA knowledge base**	“climate change” OR climate OR “climate extreme” OR “climate variability” OR flood OR flooding
**Greylit.org**	“climate change” AND sanitation OR flooding AND sanitation OR flood AND sanitation OR “climate change” AND wastewater OR “climate change” AND “waste water” OR sanitation
**Opengrey.eu**	“climate change” AND sanitation OR climate AND OR “climate change” AND wastewater OR “climate change” AND “waste water” OR sanitation OR “climate change” AND sewer OR “climate change” AND transport
**IRC wash resources**	“climate change” + Filter: Sanitation<br>Flood + Filter: Sanitation
**Who toolkit on climate change**	Filter: Climate resilient WASH
**World bank Open Knowledge Repository**	Sanitation AND "climate change" + Filter en<br>sewer AND "climate change" + Filter en<br>wastewater AND "climate change" + Filter en<br>transport AND “climate change” + Filter en
**ODI Database**	Sanitation Database Filter climate change
**Iied publication library**	Sanitation + Filter climate change<br>Sanitation + Filter: Urban

Supplemental Information, Table S3. Appraisal framework for evaluating the quality of reporting and relevance of evidence for the included studies[1]

Topic	Criteria	Questions	Notes on scoring (out of 1)
Quality of reporting	1. Objectives	Were the objectives and purpose of the study described?	0 if neither item below is included, 0.5 if one is included, and 1 if both items are included<br>• overall purpose<br>• objectives
	2. Context	Was sufficient detail provided on the context and setting of the study?	0 if neither item below is included, 0.5 if one is included, and 1 if both items are included<br>• description of sanitation system (e.g., technologies, components, services, technical status of infrastructure)<br>• details of climate change projection or extreme weather event
	3. Data collection	Was sufficient detail provided the data collection method and procedures?	0 if no information, 0.5 if partial detail is provided, and 1 if sufficient detail is provided.
	4. Analysis	Was sufficient detail provided on analytical methods used in the study?	0 if no information, 0.5 if partial detail is provided, and 1 if sufficient detail is provided.
	5. Interpretation	Is there a discussion and interpretation of the main findings?	0 if no information, 0.5 if discussion and interpretation is incomplete, and 1 if a complete discussion and interpretation is provided.
	6. Limitations	Were study limitations described?	0 if no information, 0.5 if limitations are incomplete, and 1 if full limitations are described.
	7. Conclusions	Were stated conclusions and implications within the scope of the study design and grounded in the data presented?	0 if conclusions are not stated or beyond the scope, 0.5 if partly beyond the scope, and 1 if conclusions are within the scope of the study.
Relevance and generalisability of evidence	8. Strength of evidence based on study method	How can the provided evidence presented in the results be classified?	0 if expert consultation, 0.5 if reported or modelled evidence, and 1 if empirical evidence.
	9. Spatial scale and generalisability of impacts on sanitation system	What is the spatial scale and generalisability of the presented evidence?	0 if study was very context specific with limited generalisability (e.g., impacts on sanitation system were dependent on specific project/interventions design), 0.5 if study was context specific (in terms of climate impacts and sanitation systems) but results that are transferable to similar contexts, and 1 if study had global scale or case independent approach.
	10. Temporal scale and generalisability of climate effect	What is the temporals scale and generalisability of the studied climate effect?	0 if study presented evidence linked to a single occurrence of an extreme weather event, 0.5 if study presented evidence linked to multiple occurrences of similar extreme weather events, and 1 if study presented impacts linked to a long-term observation or (projected) climate change trend.

[1] Criteria 1-7 are based on the quality appraisal framework used by Venkataramanan, V., A.I. Packman, D.R. Peters, D. Lopez, D.J. McCuskey, R.I. McDonald, W.M. Miller and S.L. Young (1) which has been adapted to reflect the requirements of the review topic and included study types.

References

Venkataramanan, V., Packman, A.I., Peters, D.R., Lopez, D., McCuskey, D.J., McDonald, R.I., Miller, W.M. and Young, S.L. 2019. A systematic review of the human health and social well-being outcomes of green infrastructure for stormwater and flood management. *Journal of Environmental Management.* **246**, pp.868-

Supplemental Information, Table S4. Results of relevance of evidence and quality of reporting evaluation for all included studies

Doc ID	Reference	Literature type	Country category	Objectives	Context	Data collection	Analysis	Interpretation	Limitations	Conclusions	Type of evidence	Spatial scale/ generalisability of impact	Temporal scale/ generalisability of climate effect	Total score
Non-sewered sanitation														
1	Abdellatif et al. (2014)	Journal article	High-income	0.5	1	1	1	1	0.5	1	0.5	0.5	1	8
2	Abdellatif et al. (2015a)	Journal article	High-income	0.5	1	1	1	1	1	1	0.5	0.5	1	8.5
3	Abdellatif et al. (2015b)	Journal article	High-income	0.5	1	1	1	1	0.5	1	0.5	0.5	1	8
4	Abdellatif et al. (2015c)	Journal article	High-income	0.5	1	1	1	1	0	1	0.5	0.5	1	7.5
5	Abdulla and Farahat (2020)	Journal article	Upper-middle income	0	1	1	1	1	0	1	1	0.5	1	7.5
6	Adin et al. (1985)	Journal article	High-income	0.5	0.5	1	1	1	0	1	0.5	0.5	1	7
8	Allouche (2006)	Conference proceedings	High-income	0.5	0.5	0.5	0	0.5	0	0	0.5	0.5	0.5	3.5
9	Aralp et al. (2016)	Conference proceedings	High-income	0	0.5	0	0	0.5	0	0.5	0.5	0.5	0	2.5
11	Bendel et al. (2013)	Journal article	High-income	0.5	1	1	1	1	1	1	0.5	0.5	1	8.5
12	Budd et al. (2020)	Journal article	High-income	1	1	1	1	1	0	0	1	0.5	1	7.5
13	Budicin (2016)	Grey literature	High-income	0.5	1	1	1	1	1	1	0.5	0.5	0.5	8
14	Butler et al. (2007)	Journal article	High-income	0.5	1	1	1	1	0	1	1	0.5	1	8
15	Cahoon and Hanke (2019)	Journal article	High-income	0.5	1	1	1	1	0	1	0.5	0.5	1	7.5
16	Cao et al. (2020)	Journal article	High-income	0.5	1	1	0.5	1	0	1	0.5	0.5	1	7
17	Chappelle et al. (2019)	Grey literature	High-income	0.5	1	1	1	1	0	1	0.5	0.5	0.5	7
18	Danilenko et al. (2010)	Grey literature	multi-country	0.5	0.5	1	0.5	1	0	1	0.5	1	0.5	6.5
20	Davis and Bursztynsky (1980)	Journal article	High-income	0.5	1	0.5	1	1	0	1	1	0.5	0	6.5
22	Draude et al. (2019)	Journal article	High-income	0.5	0.5	1	1	1	1	1	1	0.5	0.5	8
23	Flood and Cahoon (2011)	Journal article	High-income	0.5	0.5	1	1	1	0	1	0.5	0.5	1	7
24	Fortier and	Journal	High-	1	1	1	1	1	1	1	0.5	0.5	1	9

	Mailhot (2015)	article	income											
25	Gamerith et al. (2012)	Conference proceedings	High-income	0.5	1	1	0.5	0.5	0	0	0.5	0.5	1	5.5
26	Gooré Bi et al. (2015)	Journal article	High-income	1	1	1	1	1	0.5	1	1	0.5	1	9
27	Govere et al. (2010)	Journal article	Lower-middle income	1	1	1	1	1	0	1	0.5	0.5	0.5	7.5
31	Hlodversdottir et al. (2015)	Journal article	High-income	1	1	1	1	1	0	1	1	0.5	1	8.5
34	Hwang and Oleszkiewicz (2007)	Journal article	High-income	0.5	1	1	1	1	0	1	0.5	1	1	8
36	Kenward et al. (2013)	Grey literature	High-income	0.5	0.5	1	0.5	0.5	0.5	1	0.5	0.5	0	5.5
37	Kenward et al. (2016)	Grey literature	High-income	0.5	0	0.5	0	0.5	0	0.5	0.5	0.5	0.5	3.5
38	Kleidorfer et al. (2009)	Grey literature	High-income	0.5	1	1	1	0.5	0.5	1	0.5	1	1	8
39	Kleidorfer et al. (2014)	Conference proceedings	High-income	1	1	1	1	1	0.5	1	1	0.5	1	9
40	Langeveld et al. (2013)	Journal article	High-income	0.5	1	1	1	1	0	1	1	0.5	1	8
42	Mines Jr et al. (2007)	Journal article	High-income	0.5	1	1	1	1	0	1	0.5	0.5	1	7.5
44	Nie et al. (2009)	Journal article	High-income	0.5	1	1	1	1	1	1		0.5	1	8
45	Nilsen et al. (2011)	Journal article	High-income	0	0.5	1	1	1	0.5	1	0.5	0.5	1	7
46	Noi and Nitivattananon (2015)	Journal article	Lower-middle income	0.5	0.5	1	0.5	1	1	1	0.5	0.5	1	7.5
48	Pang et al. (2015)	Journal article	Upper-middle income	1	1	1	1	1	0.5	0	1	1	1	8.5
49	Plosz et al. (2009)	Journal article	High-income	1	1	1	1	1	0	1	1	0.5	1	8.5
52	Rizk and Bhattarai (2014)	Conference proceedings	High-income	0.5	1	0	0	0.5	0	0.5	0.5	1	0	4
53	Sanders (1997)	Journal article	High-income	1	0.5	1	0	1	0	1	0.5	0.5	0	5.5
54	Semadeni-Davies et al. (2008)	Journal article	High-income	1	1	1	1	1	0.5	1	0.5	0.5	1	8.5
55	Shorney (1994)	Journal article	High-income	0	0.5	0	0	0.5	0	0.5	0.5	0.5	0	2.5
58	Tait et al. (2008)	Journal article	High-income	1	1	1	1	1	0	1	0.5	0.5	1	8
59	Takamatsu et al. (2014)	Conference paper	High-income	1	0.5	0.5	0.5	1	0	0.5	0.5	0.5	0	5
60	Teichmann et al. (2019)	Conference paper	High-income	0	0	0	0	0.5	0	0.5	0.5	0.5	0.5	2.5

61	The World Bank (2018)	Grey literature	High-income	1	0	0	1	1	0	1	0.5	0.5	0.5	5.5
63	Wood et al. (2015)	Conference paper	High-income	1	0.5	1	1	0.5	0	0.5	0.5	0.5	1	6.5
64	Zamanian et al. (2020)	Conference paper	High-income	0.5	0.5	0	0	0.5	0	0.5	0.5	0.5	0.5	3.5
Onsite sanitation														
19	Cooper et al. (2016)	Journal article	High-income	1	1	1	1	1	1	1	1	0.5	1	9.5
29	Heath et al. (2012)	Journal article	Lower-middle income, Low income	1	1	1	1	1	1	1	0.5	0.5	1	9
32	Hoque et al. (1989)	Journal article	Lower-middle income	0	1	1	1	1	0	0	0.5	0	0.5	5
35	Kazi and Rahman (1999)	Conference paper	Lower-middle income	0.5	0.5	0.5	0.5	0.5	0	0.5	0.5	0.5	0.5	4.5
43	Morales et al. (2015)	Journal article	High-income	1	1	1	1	1	1	1	0.5	0.5	1	9
47	O'Driscoll et al. (2014)	Journal article	High-income	1	1	1	1	1	0.5	1	1	0.5	1	9
55	Shimi et al. (2010)	Journal article	Lower-middle income	1	1	1	1	1	0	1	0.5	0.5	0.5	7.5
62	Viraraghavan (1977)	Journal article	High-income	0	1	1	1	1	0	1	1	0.5	1	7.5
Mixed sanitation systems														
7	Alam et al. (2015)	Grey literature	Lower-middle income	1	0.5	1	0.5	1	0	1	0.5	0.5	1	7
33	Howard and Bartram (2010)	Grey literature	Global	1	1	1	0.5	1	0.5	1	0	1	1	8
18	Clemenz et al. (2020)	Journal article	Lower-middle income	1	1	1	1	1	0.5	1	0.5	0.5	1	8.5
41	McGill et al. (2019)	Journal article	Upper-middle income	1	1	1	1	1	0.5	1	1	0.5	1	9
51	Purwar et al. (2020)	Journal article	Lower-middle income	1	1	1	1	1	1	1	0.5	0.5	0	8
Transport studies														
65	Alfaro Marolo et al. (2009)	Journal article	High-income	1	1	1	1	1	0	1	0.5	0.5	1	8
66	Alhassan and Ben-Edigbe (2011)	Journal article	Upper-middle income	0.5	1	0.5	1	1	0.5	1	1	0.5	0.5	7.5

67	Anarde et al. (2018)	Journal article	High-income	1	1	1	1	1	0.5	1	0.5	0.5	1	8.5
68	Aursand et al. (2013)	Conference paper	High-income	0.5	0.5	0.5	1	1	0	0.5	0.5	0.5	1	6
69	Balakrishnan et al. (2020)	Journal article	High-income	1	0.5	1	1	1	0.5	1	1	0.5	0	7.5
70	Bíl et al. (2015)	Journal article	High-income	0.5	1	1	1	0.5	1	1	0.5	0.5	0.5	7.5
71	Bilodeau et al. (2015)	Conference paper	High-income	0.5	1	1	1	1	0	1	0.5	0.5	1	7.5
72	Bucar and Hayeri (2020)	Journal article	High-income	1	1	1	1	1	0	1	0.5	0.5	0.5	7.5
73	Chang et al. (2009)	Conference paper	High-income	1	1	1	1	1	0	1	0.5	0.5	1	8
74	Chang et al. (2010)	Journal article	High-income	1	1	1	1	1	1	1	0.5	0.5	1	9
75	Chen and Zhang (2014)	Conference paper	High-income	1	0.5	0.5	1	1	0	1	1	0.5	0.5	7
76	Diakakis et al. (2020)	Journal article	High-income	1	1	1	1	1	0	1	1	0.5	0	7.5
77	Ebuzoeme and Divine-Favour (2015)	Journal article	Lower-middle income	1	1	1	1	0.5	0	0.5	0.5	0.5	0.5	6.5
78	Elshaer et al. (2019)	Journal article	High-income	0.5	1	1	1	1	0.5	1	0.5	1	1	8.5
79	Friedrich and Timol (2011)	Journal article	Upper-middle income	1	1	1	1	1	1	1	0.5	0.5	1	9
80	Hemed et al. (2020)	Conference paper	Lower-middle income	0.5	0.5	0.5	1	0.5	0	0.5	1	0.5	1	6
81	Ismail and Ghani (2017)	Conference paper	Upper-middle income	0.5	0.5	0.5	0.5	0.5	0	0.5	0.5	0.5	0.5	4.5
82	Jacobs et al. (2018)	Journal article	High-income	1	0.5	1	1	1	1	1	0.5	0.5	1	8.5
83	Lu and Peng (2011)	Journal article	High-income	1	0.5	1	1	1	0	1	0.5	0.5	1	7.5
84	Mallick et al. (2018)	Journal article	High-income	1	0.5	1	1	1	0.5	1	0.5	0.5	1	8
85	Mallick et al. (2014)	Journal article	High-income	0.5	1	1	1	0.5	0	1	0.5	0.5	1	7
86	Mills et al. (2009)	Journal article	High-income	1	1	1	1	1	1	1	0.5	0.5	1	9
87	Mitsakis et al. (2014a)	Journal article	High-income	1	1	0.5	1	1	0	1	0.5	0.5	0	6.5
88	Mitsakis et al. (2014b)	Journal article	High-income	1	1	1	1	1	0	1	0.5	0.5	0	7
89	Mndawe et al. (2015)	Journal article	Lower-middle income	0.5	1	1	1	1	0	1	0.5	0.5	1	7.5
90	Mosqueda et al. (2007)	Conference paper	High-income	1	0.5	0.5	0.5	0.5	0	0.5	1	0.5	0	5

91	Praharaj et al. (2021)	Journal article	High-income	1	1	1	1	1	1	1	0.5	0.5	1	9
92	Pyatkova et al. (2019)	Journal article	High-income	1	1	1	1	1	0.5	1	0.5	0.5	1	8.5
93	Qiao et al. (2020)	Journal article	High-income	1	1	1	1	1	1	1	0.5	0.5	1	9
94	Read and Reed (2013)	Conference paper	High-income	0.5	0.5	0	0	0.5	0.5	0.5	0.5	0.5	0	3.5
95	Shao et al. (2017)	Journal article	High-income	0.5	0.5	1	1	1	0	1	0.5	0.5	1	7
96	Stoner et al. (2019)	Journal article	High-income	1	1	1	1	1	1	1	0.5	0.5	1	9
97	Suarez et al. (2005)	Journal article	High-income	0.5	1	1	1	1	0.5	1	0.5	0.5	1	8
98	Sultana et al. (2016a)	Journal article	High-income	1	1	1	1	1	0	1	0.5	0.5	0.5	7.5
99	Sultana et al. (2016b)	Conference paper	High-income	1	0.5	1	0.5	0.5	0	1	1	0.5	0.5	6.5
100	Sultana et al. (2016c)	Conference paper	High-income	1	1	1	1	1	0	1	1	0.5	1	8.5
101	Thiam et al. (2013)	Conference paper	High-income	1	1	1	0.5	0.5	0	1	0.5	0.5	1	7
102	Tighe et al. (2008)	Journal article	High-income	1	1	1	1	1	0.5	1	0.5	0.5	1	8.5
103	Ying and Abdul Ghani (2019)	Journal article	Upper-middle income	0	1	1	1	1	0	1	1	0.5	0.5	7
104	Zhang et al. (2008)	Journal article	High-income	0.5	1	1	1	1	1	1	1	0.5	0	8

Supplemental Material, Table S5. Details of included literature in the systematic review - sewered sanitation systems (n = 46)

DOC ID	STUDY	CLIMATE CHANGE EFFECT/HAZARD	SANITATION SYSTEM COMPONENT/PROCESS, *KNOWLEDGE CLUSTER*	TYPE OF EVIDENCE	COUNTRY/LOCATION	SUMMARY OF MAIN RESULTS	AGGREGATED QUALITY SCORE
1	Abdellatif, M., W. Atherton and R. Alkhaddar (2)	Increase in rainfall intensity and frequency	Combined sewer system (simulation for entire urban catchment area of Windermere urban drainage area)<br><br>*Primarily engineering*	Quantitative – modelled evidence (potential impact of CC on sewer system assessed using InfoWorks CS Software and applying two emission scenarios (A1F1, B1) and three global circulation models (HadCM3, CSIRO, CGCM3)	United Kingdom/North-western England (Windermere urban drainage area)	Results for annual and bathing season overflow volumes, spilling frequency and pollutant concentrations (BOD and NH3) in the 2080s varied for the different GCMs and emission scenarios.	7.5
2	Abdellatif, M., W. Atherton, R. Alkhaddar and Y. Osman (3)	Increase in rainfall intensity and frequency	Combined sewer system (simulation for entire urban catchment area of Windermere urban drainage area)<br><br>*Primarily engineering*	Quantitative – modelled evidence (derivation of future rainfall from UKCP09 weather generator, applied to existing urban drainage system (sewer system modelled using InfoWorks CS Software), use of two emission scenarios (A1F1, B1)	United Kingdom/North-western England (Windermere urban drainage area)	Results showed an increase of flood volume from overflowing inspection chambers and increased risk of basement flooding during winter.<br>Under the worst-case scenario projected to occur in the 2080s, the number of flooded inspection chambers was projected to increase by up to 26% and total flood volume to increase by 40% whereas the design storm was only projected to increase by 15%. This suggested a non-linear relation between the cause of flooding and its implications.<br>During summer season a decrease in flood volume was projected	8
3	Abdellatif, M., W. Atherton, R. Alkhaddar and Y. Osman (4)	Change in rainfall pattern (increase and decrease of rainfall/design storms)	Sewerage system of Wigan catchment made up of mixture of combined, separate and partially separate sewers serving residential population of 190,942<br><br>*Primarily engineering*	Quantitative – modelled evidence (potential impact of CC on sewer system assessed applying two emission scenarios (A1F1, B1) and three global circulation models (HadCM3, CSIRO, CGCM3)	United Kingdom/North-western England (Wigan urban drainage area)	Authors assessed impact on CC on drainage system based on change in inspection chamber flood volumes, surcharge in sewers and number of buildings at risk of flooding<br>For all three GCMs under scenario A1F1 the results suggested the number of inspection chamber flooding and number of surcharged sewers	7.5

DOC ID	STUDY	CLIMATE CHANGE EFFECT/HAZARD	SANITATION SYSTEM COMPONENT/PROCESS, *KNOWLEDGE CLUSTER*	TYPE OF EVIDENCE	COUNTRY/LOCATION	SUMMARY OF MAIN RESULTS	AGGREGATED QUALITY SCORE
						(and consequently properties at risk of flooding) in the winter season. Under scenario B1 and in the summer season under both scenarios all three implications are thought to decrease	
4	Abdellatif, M., W. Atherton, R.M. Alkhaddar and Y.Z. Osman (5)	Change in rainfall pattern: Increase of precipitation during winter and decrease of rainfall/design storms during summer	19 combined sewer overflows (CSOs) to receiving waters of Crewe urban drainage catchment area (combined sewer system serving a population of approx. 90,484.<br><br>*Primarily engineering*	Quantitative – modelled evidence (potential impact of CC on sewer system assessed applying two emission scenarios (A1F1, B1) and three global circulation models (HadCM3, CSIRO, CGCM3)	United Kingdom/North-western England (Crewe urban drainage area)	Authors quantified the spilling volume, frequency and duration of 19 CSOs in the catchment under different CC scenarios.<br>For the 2080s the simulation showed an increase for all three parameters under the high emissions scenario (up to 37% in total spill volume, 32% in total spill duration and 12% in spill frequency under the HadCM3 model). Under the B1 low emission scenario the system was expected to cope with CC impacts and a reduction in spill volume, frequency and duration was projected.<br>Projected decrease of rainfall during the summer (bathing season) led to reduction of spill volume during that season but lower water levels and dilution in receiving waters could still pose a pollution threat.	7
5	Abdulla, F. and S. Farahat (6)	Increase in temperature<br>Reduction of rainfall	WWTP (Central Irbid WWTP located in north-west of Irbid with design flow rate of 11,300m^3/day<br><br>*Primarily engineering*	Quantitative – modelled evidence (potential impact of CC on performance of WWTP were assessed applying the statistical downscaling model and the HadCM3 global climate model for two emission scenarios (A2 and B2) in conjunction with a wastewater simulation model (STOAT) for	Jordan/Irbid	During the summer months, increase in (inflow) temperature combined with no change in precipitation resulted in increase of BOD (4.2-6 mg/l), COD (>4 mg/l), and TSS (up to 1.5 mg/l) removal<br>During the winter months an increase in (inflow) temperature combined with reduction of precipitation indicated reduction of BOD (6-8 mg/l), COD (up to 6 mg/l) and TSS (up to 2.5 mg/l) removal	6.5

DOC ID	STUDY	CLIMATE CHANGE EFFECT/HAZARD	SANITATION SYSTEM COMPONENT/PROCESS, *KNOWLEDGE CLUSTER*	TYPE OF EVIDENCE	COUNTRY/LOCATION	SUMMARY OF MAIN RESULTS	AGGREGATED QUALITY SCORE
				periods of 2050-2065 and 2080-2099			
6	Adin, A., E.R. Baumann and F.D. Warner (7)	Increasing temperature	One standard-rate trickling filter WWTP plant with capacity of 0.5 mgd and 0.17 BOD_5/capita/day<br><br>*Primarily engineering*	Quantitative – empirical evidence (measurements at plant)	USA/Nevada	Establishes general relationship between moderate temperature changes of wastewater in plant (temperature 9-22 degrees).<br>BOD5 and SS removal more efficient in higher temperatures	6.5
8	Allouche, E.N. (8)	Hurricanes and other flooding mechanisms	Sewer systems in urban areas of Slidell, LA and Charles, LA<br><br>*Primarily engineering*	Qualitative – Reported data (Investigation into impact on Hurricane Andrew (1992), 1997 Red River flood and preliminary findings for investigation into Hurricane Katrina and Rita)	USA/Golf coastline	Fluctuation in pressure (internal/external) on sewer pipes during and after flooding event can lead to cracking of pipes, inflow of soil particles and ultimately ground/road collapse<br>Raw sewage pumped into lake as result of flooding of WWTP (for ten 10 days)<br>Uprooting of trees led to damage of sewer pipes<br>Damage during recovery efforts (e.g., damage to sewer drains during re-erecting of electricity poles)	3
9	Aralp, C.L., J.J. Scheri and K. O'Sullivan (9)	Extreme weather event	Two wastewater pumping stations with peak pumping capacities of 17.5 MGD and 3.0 MGD respectively<br><br>*Primarily engineering*	Qualitative – Reported data (Impacts of Superstorm Sandy)	USA/New Jersey	Inundation of wastewater pumping and treatment facility<br>Electricity outages leading to pump failures<br>Pumping station inaccessible due to flooding	2
11	Bendel, D., F. Beck and U. Dittmer (10)	Change in rainfall pattern	Combined sewer network of provincial town with 9,900 residents<br><br>*Primarily engineering*	Quantitative – modelled evidence (precipitation data was generated by the stochastic precipitation generator NiedSim-Klima and applied to idealised combined sewer model)	Germany/Baden Würtenberg	Total annual precipitation was not assumed to change substantially but trend towards shorter and more intensive events will lead to an increase in the frequency of overflow events<br>Annual CSO volumes were predicted to decrease	8
12	Budd, E., R.W. Babcock, Jr., D. Spirandelli, S. Shen and A. Fung (11)	Sea level rise	Separate sewer network in downtown Honolulu (53,116 m total length op pipes)	Quantitative – modelled evidence	USA/Honululu, Hawaii	Sea-level rise led to increased groundwater level and therefore increased groundwater infiltration into pipes (up to 50% increase in	6.5

DOC ID	STUDY	CLIMATE CHANGE EFFECT/HAZARD	SANITATION SYSTEM COMPONENT/PROCESS, *KNOWLEDGE CLUSTER*	TYPE OF EVIDENCE	COUNTRY/LOCATION	SUMMARY OF MAIN RESULTS	AGGREGATED QUALITY SCORE
			Primarily engineering			average dry weather flow by 2100). Variation in in tide ranges affected the normal diurnal flow variation between 3-10%	
13	Budicin, A.N. (12)	Prolonged dry periods drought	Wastewater treatment plant serving a population of 293,717 people<br><br>*Primarily engineering*	Quantitative – empirical evidence (analysis of WWTP data series and drought data from 2007-2015)	USA/California	Study found decreased WW flow rates and increased TIN concentrations as result of drought and related water conservation. Main impact on treatment process was found to be requirement for increased denitrifications capacity of activated sludge treatment.	7.5
14	Butler, D., B. McEntee, C. Onof and A. Hagger (13)	Increase in rainfall intensity and frequency	Sewer storage tank in North London<br><br>*Primarily engineering*	Quantitative – modelled evidence (comparison of current and future storage tank performance applying the Hadley regional climate model)	United Kingdom/London	Model predicted a 35% increase of storm events resulting in filling of the tank and an average of 57% of increase in storage required for 2080	7
15	Cahoon, L.B. and M.H. Hanke (14)	Increase in rainfall intensity and frequency<br>Sea-level rise<br>Temperature variations	19 coastal wastewater treatment plants (connected to separate sewer systems)<br><br>*Primarily engineering*	Quantitative – Empirical data (Daily flow data from four central treatment systems)	USA/North Carolina	Author investigated impact of temperature, rainfall and sea-level on inflow and infiltration. Rainfall intensity and high tides were linked to increased inflows into the wastewater treatment plants.	7
16	Cao, A., M. Esteban and T. Mino (15)	Sea level rise	Three astewater treatment plants (connected to combined and separate sewer systems). Design capacities: are 433,000 m^3/day (population of 716,192), 222,000 m^3/day(population of 308,531) , and 12,000 m^3/day (population of 38,100 people)<br><br>*Primarily engineering*	Qualitative – Reported evidence (experience from WWTP staff with land subsidence was used as proxy for future SLR)	Japan/Tohoku region	Risk of seawater intrusion into discharge pipe was reported. Ability to discharge treated wastewater by gravity would be dependent on level SLR and tidal cycle.<br>Saltwater intrusion into treatment system were reported as concern as pump motors and aeration tanks were not designed to cope with high-salinity water.<br>Rising groundwater (GW) levels lead to increased infiltration into sewer system and therefore reduced capacity of plants and exacerbated pipe corrosion.	6.5

DOC ID	STUDY	CLIMATE CHANGE EFFECT/HAZARD	SANITATION SYSTEM COMPONENT/PROCESS, *KNOWLEDGE CLUSTER*	TYPE OF EVIDENCE	COUNTRY/LOCATION	SUMMARY OF MAIN RESULTS	AGGREGATED QUALITY SCORE
						Concerns were reported about buoyancy caused by raised GW level and potential damage to pipes and resulting spillage of sewage into GW	
17	Chappelle, C., H. McCann, D. Jassby, K. Schwabe and L. Szeptycki (16)	Drought and declining rainfall	Wastewater collection and treatment systems in California<br><br>*Primarily engineering*	Mixed - Reported and expert consultation (online survey with 133 wastewater agencies and workshop with wastewater managers)	USA (California)	Reduced influent flows at WWTPs increases in biological oxygen demand (BOD), nutrient concentration, ammonia loading, total suspended solids (TSS), solids, and salinity (TDS) in wastewater influent leading to challenges/less effective treatment<br>Corrosive influent damaged equipment in treatment plants<br>Build-up of solids in sewer pipes due to declined flow rates<br>Increased generation of acids in the collection system leading to increased corrosion and reduced lifetime of infrastructure	6.5
18	Danilenko, A., E. Dickson and M. Jacobsen (17)	Increased rainfall intensity<br>Sea-level rise<br>Increased occurrence of drought	Wastewater collection and treatment systems<br><br>*Primarily engineering*	Mixed – Reported (questionnaire sent to 20 water utilities but only three responses were specific about sanitation)	USA/Seattle, New York<br>Australia/Melbourne[a]	*Increased rainfall intensity:* Changes to infiltration rates in sewer systems, increased risk of pipe failure and collapse due to changed soil moisture levels and associated subsidence<br>Increased incidence of sewer overflows<br>*Sea-level rise:* Combination of high-sea levels and heavy rains causing sewer back-up and extensive flooding<br>Increased salinity of wastewater due to increased saline infiltration<br>Drought: Increased potential for corrosion and odour in sewerage network due to increased sewage concentrations and declined flow rates	6
20	Davis, J.A. and T.A. Bursztynsky (18)	Prolonged dry periods	Wastewater treatment facilities	Quantitative – modelled and empirical data	USA/California	Authors investigated the effect of water conservation measures (as response to drought) on the	5.5

DOC ID	STUDY	CLIMATE CHANGE EFFECT/HAZARD	SANITATION SYSTEM COMPONENT/PROCESS, *KNOWLEDGE CLUSTER*	TYPE OF EVIDENCE	COUNTRY/LOCATION	SUMMARY OF MAIN RESULTS	AGGREGATED QUALITY SCORE
			Primarily engineering			performance of WWTPs. Authors concluded that there was no major harmful effect (i.e., siltation, solid deposits) from reduced flow rates (up to 20% decrease) in sewers and aerated grit chambers. Velocity-controlled grit chambers were thought to be very sensitive to flow reduction. Further, the study suggested a risk of reduced treatment efficiency due to increase pollutant concentrations	
22	Draude, S., E. Keedwell, R. Hiscock and Z. Kapelan (19)	Prolonged dry periods followed by rainfall event	Sewer systems of Cardiff and Merthyr valleys<br><br>*Primarily engineering*	Quantitative – Empirical data (Statistical analysis of data on sewer blockages and rainfall data)	United Kingdom/Wales (Cardiff and Merthyr Valleys)	Authors linked a higher probability of blockages with a preceding higher consecutive number of dry days	7
23	Flood, J.F. and L.B. Cahoon (20)	Sea level rise and high intensity rainfall event	Five coastal wastewater treatment plants connected to separate sewer system<br><br>*Primarily engineering*	Quantitative – Empirical data (Daily flow data from four central treatment systems)	USA/North Carolina	Authors linked increase in daily flow at WWTPs (caused by inflow and infiltration into) separate system with high intensity rainfall events and periods of high tide	6.5
24	Fortier, C. and A. Mailhot (21)	Change in rainfall pattern	30 selected combined sewer overflow outfalls located in 16 municipalities in southern Quebec<br><br>*Primarily engineering*	Quantitative – modelled data	Canada/Southern Quebec	Projected CSO frequency and duration under CC scenario showed only minor changes in frequency and duration with seasonal variations	8.5
25	Gamerith, V., J. Olsson, D. Camhy, M. Hochedlinger, P. Kutschera, S. Schlobinski and G. Gruber (22)	Decrease of rainfall intensity in summer Increase of rainfall intensity in winter	Combined sewer system in Linz urban catchment (downtown Linz drained mainly by combined sewers and the 39 neighbouring communes have both combined and separate systems installed). Total storage volume of the systems is approx. 115,000 m^3	Quantitative – modelled data	Austria/Linz	Results suggested an increase of total CSO volume by 17%	5

DOC ID	STUDY	CLIMATE CHANGE EFFECT/HAZARD	SANITATION SYSTEM COMPONENT/PROCESS, *KNOWLEDGE CLUSTER*	TYPE OF EVIDENCE	COUNTRY/LOCATION	SUMMARY OF MAIN RESULTS	AGGREGATED QUALITY SCORE
			Primarily engineering				
26	Gooré Bi, E., F. Monette, P. Gachon, J. Gaspéri and Y. Perrodin (23)	Increased rainfall intensity	Combined sewer systems of city of Longueuil (population 47,000 people)<br><br>*Primarily engineering*	Quantitative – Modelled data	Canada/Southern Quebec	Study investigated effects of 20% increase in rainfall intensity (CC scenario for 2041 - 2070). Authors showed that relationship between precipitation and sewer overflow variables is non-linear. Under CC conditions the model predicted an increase in discharge and sewer overflow volumes as well as an increase in pollutants being discharged to receiving water bodies	8
27	Govere, S., J. Chikazhe and C.T. Mandipa (24)	Variation in rainfall intensity	WWTP connected to structurally unsound separate sewer system. Design average dry weather flow of WWTP of 5.6 Ml/d<br><br>*Primarily engineering*	Quantitative – Empirical data	Zimbabwe/Bindura town	Study found that nutrient removal capacity of WWTP decreased during rainfall event. Study further observed a general reduction of removal capacity with increasing rainfall intensity.	7
31	Hlodversdottir, A.O., B. Bjornsson, H.O. Andradottir, J. Eliasson and P. Crochet (25)	Increase in rainfall intensity	Combined sewer system in downtown Reykjavik (model included total pipe length of around 46 km and 892 inspection chambers)<br><br>*Primarily engineering*	Quantitative – Modelled evidence	Island/Reykjavik	Authors explored current and future flood hazard in the combined sewer system. The system did not satisfy current design standards for flood control. Modelling increasing rainfall intensity led to an increase in flooded inspection chambers and overflow volumes. Flooding risk was increased for combination of CC impacts and urban development.	7.5
34	Hwang, J.H. and J.A. Oleszkiewicz (26)	Temperature extremes	WWTP (lab based experiment)<br><br>*Primarily engineering*	Quantitative – Empirical evidence (Lab based experiment with a sequencing batch reactor (SBR) in a temperature-controlled room	Canada	Study investigated effect of sharp (as opposed to gradual) decrease of temperature on nitrification effect. Results suggested that sudden temperature drop (by 10°C) affected nitrification much more than predicted and led to 20% larger decrease of nitrification than predicted by temperature correction factor. The	7.5

DOC ID	STUDY	CLIMATE CHANGE EFFECT/HAZARD	SANITATION SYSTEM COMPONENT/PROCESS, *KNOWLEDGE CLUSTER*	TYPE OF EVIDENCE	COUNTRY/LOCATION	SUMMARY OF MAIN RESULTS	AGGREGATED QUALITY SCORE
						authors argued that rapid and sharp temperature drop of wastewater inflow had been measured as a result of rapid snowmelt.	
36	Kenward, A., D. Yawitz and U. Raja (27)	Flooding from storm surge and extreme rainfall events	Sewer systems and WWTPs<br><br>*Primarily engineering*	Mixed – Reported evidence (Majority of data from reports about bypasses and overflows from wastewater utilities to EPA)	USA/East coast	Cumulative data from 6 hardest hit states showed that 6 months after Sandy at least 11 billion gallons of untreated or partially treated sewage was discharged into water bodies as a result of Hurricane Sandy.<br>Most discharges and overflows were caused by storm-surge but in Washington D.C. rainfall caused discharges and overflows<br>Severe damage to multiple treatment plants	5
37	Kenward, A., N. Zenes, J. Bronzan, J. Brady and K. Shah (28)	More intense rainfall and increased frequency of extreme events	Combined sewer systems<br><br>*Primarily engineering*	Quantitative – Reported (nationwide web search for news articles reporting on rain-related sewage overflow)	USA	Increase in rain-related sewage overflows expected	3
38	Kleidorfer, M., M. Möderl, R. Sitzenfrei, C. Urich and W. Rauch (29)	More intense and prolonged precipitation	Combined sewer systems<br><br>*Primarily engineering*	Quantitative – Modelled evidence (generation of 250 virtual case studies for case independent conclusion, use of climate change factors (CCF))	Austria/Innsbruck and case independent studies	Simulations showed increased CSOs due to increased rainfall intensities but authors also demonstrated that non-climate factors such increase of impervious area could have similar effect as CC effect	7.5
39	Kleidorfer, M., C. Mikovits, A. Jasper-Toennies, M. Huttenlau, T. Einfalt and W. Rauch (30)	More intense and prolonged precipitation	Combined sewer system of Innsbruck (total catchment area around 2,028 ha)<br><br>*Primarily engineering*	Quantitative – Modelled evidence (use of climate change factors)	Austria/Innsbruck	Authors carried out a sensitivity analysis to compare the impacts of higher rainfall intensity with the impacts of changes in paved urban areas: The model showed that an increase in rainfall intensity led to increased CSO and discharged pollutants. However, showing the relative importance of climate to non-climatic effects an increase of rainfall volume (CCF = 1.5) was modelled to have a comparable	8

DOC ID	STUDY	CLIMATE CHANGE EFFECT/HAZARD	SANITATION SYSTEM COMPONENT/PROCESS, *KNOWLEDGE CLUSTER*	TYPE OF EVIDENCE	COUNTRY/LOCATION	SUMMARY OF MAIN RESULTS	AGGREGATED QUALITY SCORE
						effect with an increase of paved areas by 80%.	
40	Langeveld, J.G., R.P.S. Schilperoort and S.R. Weijers (31)	Considers a likely combination of climate change effects: A prolonged dry period (reduced number of rainy days) followed by a high-intensity storm event	City wastewater management system (combined sewer system with 200 CSOs + 750,000 PE WWTP)<br><br>*Primarily engineering*	Quantitative – Empirical evidence (Combined analysis of long-term wastewater monitoring data and meteorological data	The Netherlands/Eindhoven	Study demonstrated potential built-up of sediments and debris in dry period leading to blockage of discharge pipes during storm event<br>WWTP experienced biological overloading<br>Oxygen levels in receiving water body dropped with recovery taking days	7
42	Mines Jr, R.O., L.W. Lackey and G.H. Behrend (32)	Change in rainfall intensity and durations	24 wastewater treatment plants with design capacities of 37,850 m^3/day or greater<br><br>*Primarily engineering*	Quantitative – Empirical evidence (Analysis of data from 24 WWTPs)	USA/Georgia	Objective of study was to determine effect of rainfall intensity on volumetric flow rate to WWTPs and to determine relationship between flow-rate and influent BOD_5 and TSS concentrations. Results suggested that BOD concentrations were diluted by larger flows due to increased inflow and infiltration. There was direct positive relationship between influent BOD load and effluent BOD concentration meaning that biological capacity of WWTP might be exceeded	7
44	Nie, L., O. Lindholm, G. Lindholm and E. Syversen (33)	More intense and prolonged precipitation	Separate and combined sewer system covering 364.30 ha catchment<br><br>*Primarily engineering*	Quantitative – Modelled evidence (simulated response of present sewer system to artificial climate change scenarios)	Norway/Fredrikstad	Authors predicted that the volume of water spilling from flooding inspection chambers would be 2-4 times and total CSO would be 1.5-8 times the increase rainfall<br>Number of flooding inspection chambers and surcharging sewers was predicted to increase substantially and irregularly	8
45	Nilsen, V., J.A. Lier, J.T. Bjerkholt and O.G. Lindholm (34)	More intense and prolonged precipitation	Sewer system in sub-catchment of Oslo (376 ha)<br><br>*Primarily engineering*	Quantitative – Modelled evidence (delta-change method used to develop time series of precipitation for the period 2071-2100. Sewer	Norway/Oslo	Simulation predicted substantial increase in CSO, flooding of inspection chamber and levels of backwater in pipes	6.5

DOC ID	STUDY	CLIMATE CHANGE EFFECT/HAZARD	SANITATION SYSTEM COMPONENT/PROCESS, *KNOWLEDGE CLUSTER*	TYPE OF EVIDENCE	COUNTRY/LOCATION	SUMMARY OF MAIN RESULTS	AGGREGATED QUALITY SCORE
				performance modelling using MOUSE sofware			
46	Noi, L.V.T. and V. Nitivattananon (35)	More intense and prolonged precipitation<br>Sea-level rise	Wastewater management system (collection and treatment)<br><br>*Primarily engineering*	Mixed – Reported evidence (Survey and FDGs)	Vietnam/ Ho-chi Minh City	Capacity of sewage network overloaded resulting in overflow to drainage network<br>Flooding of wastewater treatment site<br>Environmental pollution of surface water due to sewage network overflows into drainage system	7
48	Pang, Y., Y. Zhang, X. Yan and G. Ji (36)	Temperature variations	Constructed tidal flow wetland (lab based experiment)<br><br>*Primarily engineering*	Quantitative – Empirical evidence (Lab based experiment)	China	Results confirmed that in general removal capacity for N increases with increasing temperature. Cool weather and extreme cold periods were shown to lead to decreased efficiency of N removal (temperatures of experiment 4, 8, 12°C)	7.5
49	Plosz, B.G., H. Liltved and H. Ratnaweera (37)	Increase in winter temperature<br>More variable winter temperature	WWTP (Q_{dim} = 1,450 L/sec)<br><br>*Primarily engineering*	Quantitative – Empirical evidence (assessment of impact of air temperature on WWTP influent during winter time over 6-year period)	Norway/Oslo	Variation of temperature during winter period more relevant for performance of WWTP than average winter temperature<br>Increase in days with temporary snow melting deteriorates WWTP performance	7.5
52	Rizk, T. and R.P. Bhattarai (38)	More intense rainfall, more frequent flooding	Three WWTPs<br><br>*Primarily engineering*	Qualitative – Reported data (review of documents and WWTP records, interviews with WWTP staff)	USA/Austin, Texas	Authors summarised impacts of flood as: Flooding and total damage of WWTP, electricity failure leading to temporary plant failure, increased inflow and infiltration, damage to pumping stations, discharge of untreated and partially treated wastewater into waterways, loss of access for plant staff,	3.5
53	Sanders, D.A. (39)	Extreme rainfall event	Six selected WWTPs<br><br>*Primarily engineering*	Qualitative – Reported evidence (field visits and interviews with staff from 6 WWTP that were selected from list of 109 facilities impacted by Great Flood (1993))	USA/Missouri	Reported impacts included: Damage to WWTPs, flooding of WWTPs, inaccessibility of WWTPs due to flooded surroundings/roads, destabilisation of roads leading to challenges for supply chains, damage to wastewater wetland flora, sewer collapse and damage to roads through sewer collapse	5

DOC ID	STUDY	CLIMATE CHANGE EFFECT/HAZARD	SANITATION SYSTEM COMPONENT/PROCESS, *KNOWLEDGE CLUSTER*	TYPE OF EVIDENCE	COUNTRY/LOCATION	SUMMARY OF MAIN RESULTS	AGGREGATED QUALITY SCORE
54	Semadeni-Davies, A., C. Hernebring, G. Svensson and L.-G. Gustafsson (40)	More intense rainfall, more frequent flooding	Combined sewer system in Helsingborg serving 122,895 people<br><br>*Primarily engineering*	Quantitative – Modelled evidence, use of different storylines for urban development	Sweden/Helsingborg	Authors investigated the relative impacts of urbanisation and climate change (together and separately) on inflow volumes to WWTP, CSO frequency and volumes and nutrient transport. Authors predicted CC induced increase in CSO frequency and volumes as well as increased infiltration into sewers reducing their capacity. With different storylines on urbanisation scenarios the authors demonstrated the interrelation between climate and non-climate factors	8
55	Shorney, F.L. (41)	More intense rainfall, more frequent flooding	WWTPs<br><br>*Primarily engineering*	Qualitative – Reported evidence (no method is described but author is principal engineer of WWTP)	USA/Kansas	Wastewater was bypassed/WWTP was several days out of service<br>During flood staff had to be transported by boat to WWTP<br>Combined sewer collapses after flood/Road collapses due to old sewers collapsing.	2
58	Tait, S.J., R.M. Ashley, A. Cashman, J. Blanksby and A.J. Saul (42)	More intense and prolonged precipitation	Sewer system<br><br>*Primarily engineering*	Mixed – Expert consultation and limited modelling	United Kingdom/	Limited sewer model predicted around 20% of increase of flood volumes and CSO spills from climate factors. Climate factors and urbanisation combined led to around 40% of increase from sewage release from CSO or flooding	7.5
59	Takamatsu, M., T. Nakazato, R. Fischer, H. Satoh, F. Bonaccorso and G. Grey (43)	Extreme weather events	Wastewater treatment plants<br><br>*Primarily engineering*	Qualitative – Reported (results of study tour and knowledge exchange seminar comparing rom Japanese Tsunami with effects of super storm Sandy)[b]	USA	Flooding inside WWTP caused damaged of equipment<br>Inundation of generators and fuel pumps led to failure of emergency generators<br>Road inaccessibility led to disruption to fuel supply chains<br>Buoyancy of air and gas pipes and partially filled tanks caused further damage<br>Biological treatment was impacted by sea water inundation, lack of	4.5

DOC ID	STUDY	CLIMATE CHANGE EFFECT/HAZARD	SANITATION SYSTEM COMPONENT/PROCESS, *KNOWLEDGE CLUSTER*	TYPE OF EVIDENCE	COUNTRY/LOCATION	SUMMARY OF MAIN RESULTS	AGGREGATED QUALITY SCORE
						sewage supply and lack of oxygen supply. Sewer blockages after event because of sand and debris deposited in sewers and pump stations. Uplifting of inspection chambers causes debris and sand to enter sewers. Equipment submerged in sea water was even more damaged	
60	Teichmann, M., N. Szeligova and F. Kuda (44)	More intense rainfall, more frequent flooding	Sewer systems with poor structural and technical condition<br><br>*Primarily engineering*	Qualitative – Reported data (multiple case studies based on secondary data)	Czech Republic	Development of sinkholes due to leaking sewers and floods causing damage of road network	2
61	The World Bank (45)	Increased flooding	Wastewater management systems<br><br>*Primarily engineering*	Mixed – Reported data utilities	Japan/various location	Authors presented data on households affected by on-floor and under-floor inundation from different rainfall events	5
63	Wood, D.M., A. Roche, M. Chokshi and K. May (46)	Combination of sea-level rise, higher magnitude rainfall and extreme tides	Combined sewer system of San Francisco<br><br>*Primarily engineering*	Quantitative – Modelled data	USA/San Francisco	Simulated higher water surfaces caused compromise of combined sewer discharge facilities. Hydraulic head was not sufficient to force water through backflow prevention devices and causes potential risk of flooding at low-points in the system.	6
64	Zamanian, S., M. Rahimi and A. Shafieezadeh (47)	Extreme events leading to flooding	Sewer networks<br><br>*Primarily engineering*	Qualitative – Reported data (reported implications from past extreme events in various river basins based on six case studies)	USA/various	Reported implications from various flooding events were: sewer overflows, damage and disruptions of WWTP leading to contamination of water bodies	3

[a] questionnaire was sent to 20 water utilities world-wide but only three utilities gave information for climate change impacts on their sanitation system
[b] results related to tsunami were not included in the review

Supplemental Material, Table S6. Details of included literature in the systematic review – non-sewered sanitation systems (including road-based transport systems (n = 48)

DOC ID	STUDY	CLIMATE CHANGE EFFECT/HAZARD	SANITATION SYSTEM COMPONENT/PROCESS, *KNOWLEDGE CLUSTER*	TYPE OF EVIDENCE	COUNTRY/LOCATION	SUMMARY OF MAIN RESULTS	AGGREGATED QUALITY SCORE
19	Cooper, J.A., G.W. Loomis and J.A. Amador (48)	Increased temperatures<br>Higher groundwater table	Septic tanks (conventional and shallow Soil treatment area (STA)- lab experiment))<br><br>*Primarily engineering*	Quantitative – empirical evidence (laboratory experiment)	USA/lab experiment simulating expected climate change (CC) in the glaciated North-eastern U.S.	The effects of CC induced increased soil temperature and groundwater table on contaminant removal capacity depend on the contaminant and STA type. In the lab experiment, removal of FCB, total P and total N in shallow narrow STAs diminished under CC conditions. In contrast, total N removal in conventional STAs improved. Viral pathogens and BOD5 were well removed under present climate and climate change conditions.	9.5
29	Heath, T.T., A.H. Parker and E.K. Weatherhead (49)	Increased pluvial flooding<br>Increased flash flooding	Pit latrines, septic tanks and latrines (unspecific) data relies mainly on interviews in selected low-income areas<br><br>*Sanitation and development*	Qualitative - Reported and observed (KIIs, FGDs and field observations)	Madagascar/Antananarivo (peri-urban and informal areas)<br>Zambia/Lusaka (Chazanga and Kanyama peri-urban areas)<br>Kenya/Naivasha (peri-urban areas)	**Antananarivo**: Collapse of latrine superstructures<br>In flooded areas latrines become unusable and use of 'flying toilets' increases<br>Damage to and inundation of roads leading to travel interruption<br>**Lusaka:** Submerge of latrines<br>Increase of risk of collapsing of latrines<br>Erosion of soil around septic tanks absorption field will reduce its efficiency<br>**Naivasha:** Latrines overflow and become unstable/collapse<br>Roads become impassable	8.5
32	Hoque, B.A., S.R.A. Huttly, K.M.A. Aziz, Z. Hasan and M.Y. Patwary (50)	Increase of flooding	Double pit-pour flush latrines (n=720, approx. serving 800 hh and 4,800 people) installed as part of a rural water supply and sanitation intervention<br><br>*Sanitation and development*	Quantitative – reported evidence (regular monitoring enabled pre- and post-flood comparison)	Bangladesh/rural area 60 km from Dhaka	Drop in latrine usage after flood event<br>Damage to slabs, pits and superstructure<br>Fencing was component that suffered most damage which was the only component that was household installed and managed	4.5

DOC ID	STUDY	CLIMATE CHANGE EFFECT/HAZARD	SANITATION SYSTEM COMPONENT/PROCESS, *KNOWLEDGE CLUSTER*	TYPE OF EVIDENCE	COUNTRY/LOCATION	SUMMARY OF MAIN RESULTS	AGGREGATED QUALITY SCORE
35	Kazi, M.N. and M.M. Rahman (51)	Increased flooding (pluvial and tidal)	Single pit latrines in three areas (no sample size given)<br><br>*Sanitation and development*	Qualitative – Reported evidence	Bangladesh/Dhaka, Patuakhali and Sylhet	All sampled latrines were inundated and overflowing during times of flooding but non-climatic factors (overuse) also played a role<br>Overflow of was caused by rainfall, overuse, and rise of groundwater	4
43	Morales, I., J.A. Amador and T. Boving (52)	Increase in temperature<br>Increased precipitation	Soil-based onsite wastewater treatment systems (septic tanks)<br><br>*Primarily engineering*	Quantitative – modelled data	North-eastern USA	Moderate temperature increase (increase of 3°C degree was modelled) in moderate to warm climate zones will increase bacterial reduction in soils	8.5
47	O'Driscoll, M.A., C.P. Humphrey, Jr., N.E. Deal, D.L. Lindbo and M.A. Zarate-Bermudez (53)	Sea-level rise	Onsite Wastewater Treatment System for year round residential site<br><br>*Primarily engineering*	Quantitative – Empirical evidence (two years field study)	USA	Study found that N from OWTS is more mobile in raised groundwater table with implications for pollution of GW and surface water	8
55	Shimi, A.C., G.A. Parvin, C. Biswas and R. Shaw (54)	Increased pluvial flooding	Latrines (unspecific)<br><br>*Sanitation and development*	Quantitative – Reported evidence (Questionnaire survey including 120 households)	Bangladesh/ Goalanda Upazilla in Rajbari district	During two major flood events in1998 and 2008 about 97% of the toilets became unusable due to inundation<br>Around 90% of the population coped by either reverting to open defecation or building temporary hanging latrines (both directly into water body)<br>Some reports about household adaptation measures (raising base or increasing base height of toilet but no specification on extent to which this is happening)	7
62	Viraraghavan, T. (55)	Temperature variations/extremes	Septic tank connected to individual household<br><br>*Primarily engineering*	Quantitative – empirical evidence (measurement with test tile over 15 months)	Canada/Ottawa	Winter operation did not pose particular problems for septic tank operation. However, climate factors only played a reduced role for liquid temperatures in the tank as the system was connected to a household with domestic hot water and thus the temperature of liquids in the tank remained at an average of 16°C even during periods when the air temperature	6.5

DOC ID	STUDY	CLIMATE CHANGE EFFECT/HAZARD	SANITATION SYSTEM COMPONENT/PROCESS, *KNOWLEDGE CLUSTER*	TYPE OF EVIDENCE	COUNTRY/LOCATION	SUMMARY OF MAIN RESULTS	AGGREGATED QUALITY SCORE
						was below freezing point and the soil temperature outside the tank was 2.8°C<br>Comparatively treatment efficiency for most parameters was higher during summer and fall with higher air and soil temperature	
Studies from road-based transport knowledge cluster (n = 40)							
65	Alfaro Marolo, C., A. Ciro German, J. Thiessen Kendall and T. Ng (56)	Increase of temperature	Road embankments / pavement (section of provincial road)	Quantitative – modelled data	Canada	Study linked projected warming temperatures with permafrost degradation beneath road embankments resulting in physical damage to pavements (lateral spreading and settlement of road embankments)	7.5
66	Alhassan, H.M. and J. Ben-Edigbe (57)	Variations in frequency and intensity of rainfall and flooding	Highway section / Road network performance (section of highway straight section (two-way interurban arterial)	Quantitative – empirical evidence	Malaysia	Authors found non-linear relationship between rainfall intensity and travel parameters (capacity, loss in travel time) but general decrease of road capacity form dry to wet weather.	6.5
67	Anarde, K.A., S. Kameshwar, J.N. Irza, J.A. Nittrouer, J. Lorenzo-Trueba, J.E. Padgett, A. Sebastian and P.B. Bedient (58)	Sea-level rise / flooding from storm surges	Bridges surrounding Freeport, Texas	Quantitative – modelled data	USA/Freeport, Texas	Study found increasing probability of bridge failure with increasing storm intensity and sea-level.	8
68	Aursand, P.O., R. Evensen and B.O. Lerfald (59)	Increasing temperature/Increasing rainfall	Pavements of roads in various climatic regions of Norway	Quantitative – modelled data	Norway	Study found that the positive impacts of increased temperature (reduced frost index) outweighed the negative impacts increased precipitation. Overall, the authors predicted and expected increase in pavement life time of 13-16 %.	5.5
69	Balakrishnan, S., Z. Zhang, R. Machemehl and M.R. Murphy (60)	Extreme events/storm	Road network performance (Houston freeway network consisting of 485 freeway links)	Quantitative – empirical data	USA/Houston	Study assessed impacts of extreme event by studying extreme travel time observations. In week of hurricane highest concentration of extreme travel time observations were made but largest magnitude of extreme observations was recorded in the weeks after the hurricane. Thus studies shows the prolonged	6.5

DOC ID	STUDY	CLIMATE CHANGE EFFECT/HAZARD	SANITATION SYSTEM COMPONENT/PROCESS, *KNOWLEDGE CLUSTER*	TYPE OF EVIDENCE	COUNTRY/LOCATION	SUMMARY OF MAIN RESULTS	AGGREGATED QUALITY SCORE
						impact of extreme weather on transport (severe pavement damage or prevalent flooding)	
70	Bíl, M., R. Vodák, J. Kubeček, M. Bílová and J. Sedoník (61)	Increase in rainfall<br>Rapid snow melt	Road network performance on various roads in Czech Republic	Quantitative<br>Empirical/reported data	Czech Republic/various	Authors investigated six major natural disasters which were all induced by extreme rainfall or by rapid snowmelt and resulted in floods and landslides. Impacts were evaluated with respect to damage to road networks and decreased serviceability. During one of the events the road network serviceability was reduced by up to 30% and the ratio of network lengths was reduced to 78.4%	7
71	Bilodeau, J.P., F.P. Drolet, G. Doré and M.F. Sottile (62)	Rising temperature	Pavement	Quantitative – modelled data	Canada/Quebec	Study showed mixed impacts of increased winter temperature:<br>Positive impact by reducing pavement damage caused by frost heaves.<br>Negative impact: increase in the number of winter thaw events resulting in increasing damage of permanent deformation in the granular base and by fatigue in the asphalt concrete layer.<br>Overall reduction of life-time of flexible pavements is expected	7
72	Bucar, R.C.B. and Y.M. Hayeri (63)	Increasing precipitation<br>SLR	Road network performance in Hoboken, New Jersey (36 miles of road)	Quantitative – modelled data (Evaluated the impacts of different magnitude storm events under low and high tide on the surface transportation system with respect to VHT (vehicle hours travelled) , VMT (vehicle miles travelled) and trips completed (TC)	USA/Hoboken	Study associated disrupted links with higher tide and storm magnitudes. Comparing low and high tide scenarios showed a drop in TC of up to 2.5%, while VHT and VMT increased by up to 41% and 19% when compared to scenarios with the storm magnitudes.	7
73	Chang, H., M. Lafrenz, W. Jung II, M. Figliozzi and D. Platman (64)	Flooding (pluvial)	Road network performance in Johnson and Fanno Creek in the	Quantitative – modelled data	USA/Portland, Oregon (Johnson Creek and Fanno Creek)	Study found relatively small impact of flooding on VMT but flooding was associated with	7.5

DOC ID	STUDY	CLIMATE CHANGE EFFECT/HAZARD	SANITATION SYSTEM COMPONENT/PROCESS, *KNOWLEDGE CLUSTER*	TYPE OF EVIDENCE	COUNTRY/LOCATION	SUMMARY OF MAIN RESULTS	AGGREGATED QUALITY SCORE
			Portland metropolitan area			vehicle hour delays as result of road closures and congestion	
74	Chang, H., M. Lafrenz, I.-W. Jung, M. Figliozzi, D. Platman and C. Pederson (65)	Increase in winter precipitation	Road network performance in Johnson and Fanno Creek in the Portland metropolitan area	Quantitative – modelled data	USA/Portland, Oregon (Johnson Creek and Fanno Creek)	Study found negligible increases in VMT but substantial increase in vehicle hour delay as result of flooding	8.5
75	Chen, X. and Z. Zhang (66)	Extreme weather (hurricanes)	Pavements in District 02 in New Orleans	Quantitative – empirical data	USA/New Orleans	Authors linked pavement performance damage to sustained flooding and heavy trucking/vehicle loading during clean-up of debris	6
76	Diakakis, M., N. Boufidis, J.M. Salanova Grau, E. Andreadakis and I. Stamos (67)	Extreme precipitation / Flash flooding	Road infrastructure/bridges and road network performance	Mixed – empirical data	Greece/Mandra	Study found extensive impact of flooding with ~40% of road network being inundated or inaccessible and over 80% of river crossings suffering structural damages. Road networks were affected heavily with substantial increase in travel time and drops in vehicle speed.<br>Large portions of road were inaccessible due to missing connection links (rather than due to flooding)	6.5
77	Ebuzoeme and O. Divine-Favour (68)	Pluvial flooding	Road network performance in six communities in Awka Anambra State	Quantitative – reported evidence	Nigeria/Awka (six communities)	Survey results found road congestions and accidents as reported impacts of flooding.	6
78	Elshaer, M., M. Ghayoomi and J.S. Daniel (69)	Increased precipitation / rising groundwater levels	Pavements (three pavement cross sections)	Quantitative – modelled data	USA	Simulation showed that pavement structure loses significant structural capacity when unbound material layers were saturated.	8
79	Friedrich, E. and S. Timol (70)	Sea-level rise	Road networks (major roads within 2 m and 4 m contour along coast and river mouths)	Quantitative – modelled data	South Africa/ eThekwini Municipality	Study modelled traffic volume changes, travel time and volume to capacity ratio	8.5
80	Hemed, A., L. Ouadif, L. Bahi and A. Lahmili (71)	Increased precipitation Increased temperature	Road pavements (road section)	Quantitative – empirical data	Morocco/Province of Chefchaouen	Study showed mixed impacts of increased winter temperature:<br>Positive impact by reducing pavement damage caused by frost heaves.<br>Negative impact: increase in the number of winter thaw events resulting in increasing rutting	5

DOC ID	STUDY	CLIMATE CHANGE EFFECT/HAZARD	SANITATION SYSTEM COMPONENT/PROCESS, *KNOWLEDGE CLUSTER*	TYPE OF EVIDENCE	COUNTRY/LOCATION	SUMMARY OF MAIN RESULTS	AGGREGATED QUALITY SCORE
						performance problems and fatigue cracking of pavement Increase precipitation was associated with pavement rutting and permanent deformation due to fatigue cracking	
81	Ismail, M.S.N. and A.N.A. Ghani (72)	Flooding	Road pavements	Qualitative – Reported evidence	Malaysia/Kedah state	Study described common damages after flooding as block cracking and edge cracking	4
82	Jacobs, J.M., L.R. Cattaneo, W. Sweet and T. Mansfield (73)	Sea-level rise Flooding	Road networks	Quantitative – modelled data	USA/East Coast	Study assessed magnitude of transportation impacts from nuisance flooding under current and future climate scenario. Nuisance flooding was found to cause over 100 million hours of vehicle-hour delay annual and is simulated to substantially increase under future sea-level scenarios	8
83	Lu, Q.-C. and Z.-R. Peng (74)	Sea-level rise Flooding	Road networks in south Miami	Quantitative – modelled data	USA/Miami, Florida	Study links different scenarios of SLR to increases in travel time and road accessibility reduction	7
84	Mallick, R.B., J.M. Jacobs, B.J. Miller, J.S. Daniel and P. Kirshen (75)	Increased precipitation Increased temperature	Road pavements	Quantitative – modelled data	USA	Authors use Monte-Carlo simulation to investigate impact of different changes in temperature and precipitation on road rehabilitation. For all scenarios there will be an increase in demand for rehabilitation	7.5
85	Mallick, R.B., M.J. Radzicki, J.S. Daniel and J.M. Jacobs (76)	Various	Road pavements	Quantitative – modelled data	USA	Results predict an average pavement life decrease from 16 to 4 years over the span of 100 years (due to increase in number of 100% saturation months, number of inundation and increase in maximum pavement temperature)	6.5
86	Mills, B.N., S.L. Tighe, J. Andrey, J.T. Smith and K. Huen (77)	Various	Road pavements (multiple study sites in southern Canada)	Quantitative – modelled data	Canada/various	Study showed that increasing temperatures will lead to reduced low-temperature cracking of pavements but structures will freeze later and thaw earlier and experience higher in-service pavement temperature resulting in increased rutting. Overall CC	8.5

DOC ID	STUDY	CLIMATE CHANGE EFFECT/HAZARD	SANITATION SYSTEM COMPONENT/PROCESS, *KNOWLEDGE CLUSTER*	TYPE OF EVIDENCE	COUNTRY/LOCATION	SUMMARY OF MAIN RESULTS	AGGREGATED QUALITY SCORE
						impacts on pavement structures were found to be modest	
87	Mitsakis, E., I. Stamos, M. Diakakis and J.M.S. Grau (78)	High intensity rainfall events<br>Storm	Road networks in metropolitan area of Athens	Quantitative – empirical data	Greece/Athens	Investigated impacts of high-intensity storms with the case of the February 2013 storm in Athens. Results highlighted disruptive effects of flooding on transport network in terms of increased travel times, significant changes in routing and substantial speed drops. Specifically, analysed impacts on freight travel and showed reduced distance travelled (probably due to planned trips being cancelled or postponed)	6
88	Mitsakis, E., I. Stamos, A. Papanikolaou, G. Aifadopoulou and H. Kontoes (79)	Extreme heat	Road networks in Peloponnesus (178,734 directed roads and 70,137 nodes)	Quantitative – modelled data	Greece/ Peloponnesus	Study linked closures of roads and travel interruption to wildfires	6.5
89	Mndawe, M.B., J.M. Ndambuki, W.K. Kupolati, A.A. Badejo and R. Dunbar (80)	Increased rainfall	Road pavements (roads P434 and P443 within uMkhanyakude District Municipality	Quantitative – modelled data	South Africa/ uMkhanyakude District Municipality	Study found that CC effects on precipitations (50-year projection) will be minimal and impacts on pavement life will be negligible relative to natural weather variability.	7
90	Mosqueda, G., K.A. Porter, J. O'Connor and P. McAnany (81)	Extreme weather (hurricanes)	Bridges	Qualitative – empirical data	USA/Gulf Coast	Study investigated impact of hurricane on bridges (amongst others): Hurricane was found to damage bridges and lead to (temporary) bridge closures and related traffic interruptions. Structural damage to bridges were linked with hits from barges, containers and debris, fast-moving waters undermining bridge piers and causing flooding of control rooms of moveable bridges.	4
91	Praharaj, S., T.D. Chen, F.T. Zahura, M. Behl and J.L. Goodall (82)	Coastal flooding<br>SLR	Road network in Norfolk Virginia	Quantitative – empirical and modelled	USA/Norfolk Virginia	Study found statistically significant reduction in VHT and VMT as a result of drivers abandoning trips due to recurrent flooding	8.5
92	Pyatkova, K., A.S. Chen, D. Butler, Z.	Flooding	Road networks in city of Marbella	Quantitative – modelled data	Spain/Marbella	Study compared normal (dry) with flooded traffic scenario and	8

DOC ID	STUDY	CLIMATE CHANGE EFFECT/HAZARD	SANITATION SYSTEM COMPONENT/PROCESS, *KNOWLEDGE CLUSTER*	TYPE OF EVIDENCE	COUNTRY/LOCATION	SUMMARY OF MAIN RESULTS	AGGREGATED QUALITY SCORE
	Vojinović and S. Djordjević (83)					observed that congestion did not evolve proportionally with reduction of traffic capacity and knock-on effects on traffic systems may be revealed delayed. Locations of traffic disruption could not be directly associated with areas of closed streets which showed that congestion can be related to lost connectivity far from flooded areas.	
93	Qiao, Y., J. Santos, A.M.K. Stoner and G. Flinstch (84)	Increased precipitation Increased temperature	Road pavements	Quantitative – modelled data (modelling applied to typical pavements in three case studies in Virginia (Bristol, Richmond and Roanoke)	USA/Virginia	Simulation showed that for most cases surface degradation of asphalt-concrete pavements will increase under CC conditions.	8.5
94	Read, W. and D. Reed (85)	Extreme weather	Road network disruptions	Mixed – reported evidence	USA and Canada	Study investigated road closures and traffic disruptions during storm event. Road closures were associated with fallen trees and rocks that blocked traffic (67%), fallen powerlines and flooding (10%) and high crosswinds (6%), or due to mudslides, traffic light failures and road accidents (17%). Several bridges were closed leading to access problems for commuters. Results showed interdependence between power delivery and transportation (downed powerlines and traffic light failures caused road closures)	3
95	Shao, Z., G. Jenkins and E. Oh (86)	Decreased precipitation Increased temperature	Road pavement performance	Quantitative – modelled evidence	Australia	Results suggested that decreased precipitation will have positive impact on slow flexible pavement deterioration but increasing temperature and solar radiation causes pavement materials to expand and increases surface degradation	6.5
96	Stoner, A.M.K., J.S. Daniel, J.M. Jacobs,	Various	Road pavement performance	Quantitative – modelled evidence (modelling quantifies impact of	USA/various	Study found general trend for reduced lifetime of current pavement designs under CC	8.5

DOC ID	STUDY	CLIMATE CHANGE EFFECT/HAZARD	SANITATION SYSTEM COMPONENT/PROCESS, *KNOWLEDGE CLUSTER*	TYPE OF EVIDENCE	COUNTRY/LOCATION	SUMMARY OF MAIN RESULTS	AGGREGATED QUALITY SCORE
	K. Hayhoe and I. Scott-Fleming (87)			three different future climates on pavement performance in 24 locations across USA)		conditions but magnitude of impact was found to be dependent on pavement types and location	
97	Suarez, P., W. Anderson, V. Mahal and T.R. Lakshmanan (88)	Coastal flooding (sea-level rise)<br>Extreme weather	Road network disruptions in Boston Metro area	Quantitative – modelled evidence	USA/Boston	For 2000 – 2100 study predicted and increase in delays (80%) and trips lost (82%) under CC scenarios. Coastal floods were found to result in greater reduction in number of trips while riverine floods were associated with greater increases in VMT and VHT.	7.5
98	Sultana, M., G. Chai, S. Chowdhury and T. Martin (89)	Extreme weather/flooding	Road pavement performance	Quantitative – modelled evidence	Australia/Queensland	Study observed that a recent flooding event caused rapid increase in deterioration of the structural and surface conditions such as roughness and rutting of the pavement	7
99	Sultana, M., G. Chai, S. Chowdhury and T. Martin (90)	Increased precipitation/flooding	Road pavement performance (case study from one street in suburb in south Brisbane)	Quantitative – empirical evidence	Australia/Brisbane	Study linked flooding to increases in roughness and rutting of pavements and increased number of pavement repairs. The study concluded that flooding escalates the deterioration of the road pavements and increases the need for frequent rehabilitation	5.5
100	Sultana, M., S. Chowdhury, G. Chai and T. Martin (91)	Increased occurrence of extreme weather / flooding	Road pavement performance based on six Brisbane City Council streets	Quantitative – empirical evidence	Australia/Brisbane	Study simulated the effects of pavement saturation due to flooding. In some cases, the pavements did not regain their pre-flooding strength even after the water recedes. Thus increased frequency of extreme weather events and flooding was found to increase demand and costs of road maintenance.	7.5
101	Thiam, P.M., G. Dore and J.P. Bilodeau (92)	Increased precipitation	Road pavement performance	Quantitative – modelled evidence	Canada/Quebec	Study associated expected increase in precipitation with significant impacts on pavement performance with an approximate reduction of long-term performance by up to 15%.	6.5

DOC ID	STUDY	CLIMATE CHANGE EFFECT/HAZARD	SANITATION SYSTEM COMPONENT/PROCESS, *KNOWLEDGE CLUSTER*	TYPE OF EVIDENCE	COUNTRY/LOCATION	SUMMARY OF MAIN RESULTS	AGGREGATED QUALITY SCORE
102	Tighe, S.L., J. Smith, B. Mills and J. Andrey (93)	Increased precipitation	Road pavement performance	Quantitative – modelled evidence (based on six test sites)	Canada	Results linked climate change impacts (increased precipitation) to exacerbated pavement rutting and cracking and as a result reduced design life. However, the impacts of CC were found to be modest in relation to regional climate variation and the impacts of increased future traffic.	8
103	Ying, L.K. and A.N. Abdul Ghani (94)	Increased precipitation/	Road pavement performance	Quantitative – empirical evidence	Malaysia/Penang state	Study found that rate of deterioration of structural strength and surface conditions of pavements will increase with increasing frequency of extreme rainfall events and flooding	6
104	Zhang, Z., Z. Wu, M. Martinez and K. Gaspard (95)	Extreme weather (hurricane flooding)	Road pavement performance	Quantitative – empirical evidence (considering three pavement types)	USA/New Orleans	Study investigated impacts of flooding during and after hurricane. Damage was found to be dependent on pavement type and exposure to flood waters with asphalt-concrete (AC) pavements in low-elevations most affected (reduction of stiffness of AC layer and sub-grade)	7

Supplemental Material, Table S7. Details of included literature in the systematic review - mixed sanitation systems (n = 5)

DOC ID	STUDY	CLIMATE CHANGE EFFECT/HAZARD	SANITATION SYSTEM COMPONENT/PROCESS, *KNOWLEDGE CLUSTER*	TYPE OF EVIDENCE	COUNTRY/LOCATION	SUMMARY OF MAIN RESULTS	AGGREGATED QUALITY SCORE
7	Alam, S.S., A.J. Alam and S. Rahman (96)	Increased flooding (pluvial) caused by more intense rainfall events and more frequent/intense extreme events	Sanitation systems in two in slum settlements (mostly community toilets) connected to septic tanks or sewerage system which is poorly designed<br><br>*Sanitation and development*	Qualitative – Reported (KIIs and FGDs)	Bangladesh/Dhaka (Beguntila and Dhalpur slums)	Overflow of septic tanks<br>Open defecation as coping mechanism with flooded toilets<br>Poorly designed sewerage systems get blocked during rainfall	
33	Howard, G. and J. Bartram (97)	Increased and more intense rainfall<br>Decreased rainfall and more frequent occurrence of drought	Pit latrines, septic tanks, wastewater collection and treatment systems<br><br>*Sanitation and development*	Qualitative – Expert consultations (series of KIIs and online survey, impact/vulnerability assessment also heavily relies on literature review)	Global	**Decreased rainfall:** Soil erosion might destabilise latrine pits<br>Lack of water leading to operational challenges with septic tanks<br>Build-up of blockages and deposition of solids in sewer<br>**Increased rainfall:** Pit latrine flooding leading to contamination<br>Rising groundwater can lead to flooding of pit and contamination of shallow groundwater<br>Flooding was associated with challenges of septic tank drainfield operation and causing tank flotation as well as potential contamination from drainfield and tank flooding<br>Increase of combined sewer overflows and backflowing of sewage<br>Sea-level rise: Risk of backflow of wastewater	
18	Clemenz, N., R. Boakye and A. Parker (98)	Increased and more intense rainfall<br>Decreased rainfall and more frequent occurrence of drought	Septic tanks, sewer system in two low-income communities in Ghana (approx. 15,000 residents each)	Qualitative - Reported and observed (KIIs, FGDs and field observations)	Ghana/low-income communities in Accra	Contamination from overflowing of septic tanks and damaged sewer pipes<br>Lack of water for flushing toilets due to electricity outages	

			Sanitation and development			(some related to reduced hydro-electric potential during dry periods)
41	McGill, B.M., Y. Altchenko, S.K. Hamilton, P.K. Kenabatho, S.R. Sylvester and K.G. Villholth (99)	Decreased rainfall and more frequent occurrence of drought	Pit latrines and flush toilets connected to sewerage system in Ramotswa peri-urban town (approx.. 37,500 people)<br><br>*Sanitation and development*	Mixed – Empirical and reported evidence (KII interviews about sanitation behaviour, measurements of groundwater contamination)	Botswana/Ramotswa aquifer	During water restrictions people abandon flush toilets and use pit latrine<br>Pit latrines are thought to contribute to groundwater pollution
51	Purwar, D., R. Sliuzas and J. Flacke (100)	Flooding due to extreme weather event (typhoon)	Septic tanks, toilets connected to drains and releasing waste into swamp water and sewer connections in Catmon (27 informal settlements, approx. 5,000 hh and population of 31,137)<br><br>*Sanitation and development*	Qualitative – Reported evidence (FDGs, expert interviews and field observations)	Philippines/low-income area of Manila	Toilets became non-functional due to lack of water supply (lea to electricity failure)<br>Inundation of toilets<br>People were unable to use toilets for multiple days and opted for open defecation

Supplemental Material, Figure S1. Regional distribution of evidence

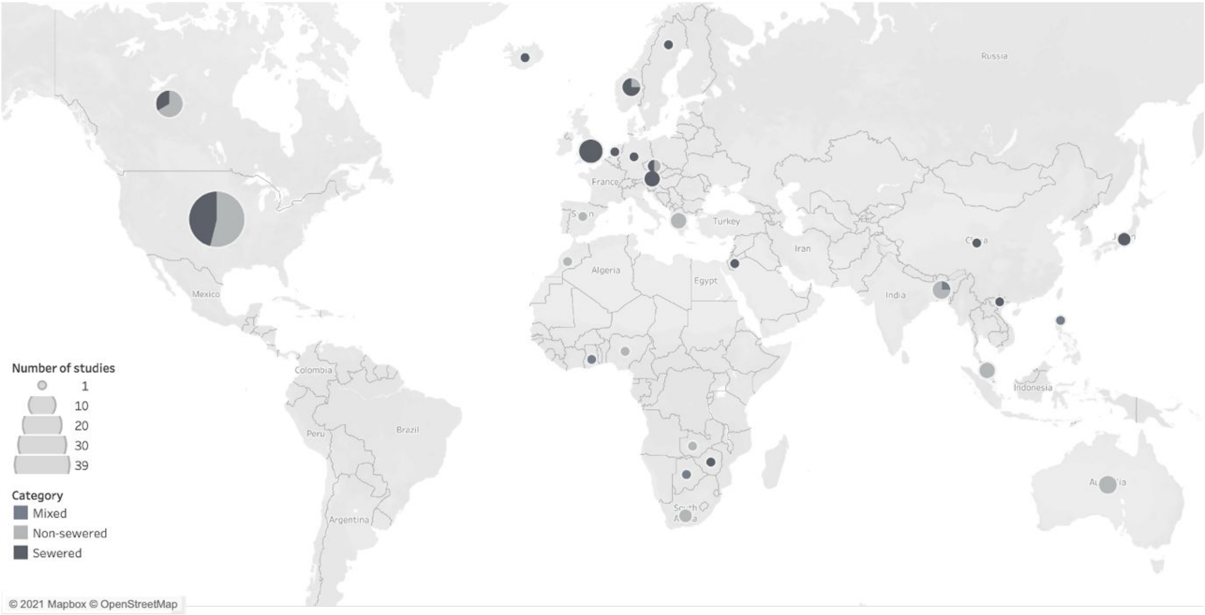

Supplemental Material, Table S8. Failure mode matrix including only results from studies scoring 7.5 (75%) or above in aggregated relevance of evidence and quality of reporting score (n = 40)

Climate change effect	Potential hazards and changes	Relevance for urban sanitation failure modes (FM) according to Peal et al. (2020)				
		FM1	FM2	FM3	FM4	FM5
		Faecal sludge not contained not emptied	Faecal sludge and supernatant not delivered to treatment	Faecal sludge and supernatant not treated	Wastewater not delivered to treatment	Wastewater not treated
More intense and prolonged precipitation / More frequent or intense storms or cyclones	High-intensity rainfall, increased flooding, erosion and landslides	Damage to pits or superstructures making latrine unusable	People 'drain' toilets during flood event	Flooding and damage to wetland flora	Increase frequency and/or spill volumes of CSO	Flooding and damage to WWTPs structure and equipment
		Pits overflow/collapse leading to faecal contamination	Structural damage to pavements		Increased risk of urban flooding (overflow of inspection chambers, flooding of basements)	Flooding of WWTP leading to temporary system failure and discharge of raw sewage
		Toilets become inundated/inaccessible (causing people to abandon toilets and revert to open defecation)	Road collapse or development of sinkholes due to destabilisation of soil caused by damaged sewers		Increased risk of pipe damage due to changed soil moisture and associated subsidence	Electricity failure leading to failure of pumps and aeration
					Changes to inflow and infiltration rates into sewer system	Road interruptions leading to disruption of site access for WWTP staff and supplies
		Electricity failure resulting in lack of water supply and non-functioning of toilets	Damage to roads infrastructure elements other than pavements (e.g., bridges)		Sewer blockages after event because of sand, debris or solid waste entering sewers and pump stations	Pollutant load exceeding biological treatment capacity of WWTP
		Inundation of drainfields	Road capacity decreases/ Increase in congestions/Travel time increases		Electricity failure leading to failure of pumps	Discharge of untreated/partially treated effluent due to overloading or bypassing of treatment
		Back-flow/overflow of sewage from septic tanks	Roads become inaccessible		Damage to sewer pumps and mains	Increased dilution of influent
		Damage to pits, septic tanks and absorption fields	Electricity failure leading to traffic light failures		Overload of sewer system resulting in overflow to drainage system	Reduced nutrient removal capacity during high intensity rainfall events (e.g., due to reduced retention time and high nutrient load)
	Contamination of and damage to surface water and groundwater supplies				Higher pollutant concentrations in receiving waters due to increase in CSO spill volumes/ frequency	Contamination of receiving water bodies due to WWTP failure
	Changes to groundwater recharge and groundwater levels	Floatation and damage of septic tanks due to high GW levels	Structural damage to pavement (destabilisation of the substrate)			Inflow and infiltration into separate systems causes higher inflow into WWTP that stretch their design capacity
		Flooding of pits from below				
		Higher groundwater pollution risk				
	More extreme winds				Uprooting of trees and replacement of damaged electricity poles leading to damage of sewer pipes	Damage to WWTP infrastructure/buildings
More variable or declining rainfall or run-off	Longer dry periods, increased frequency of occurrence of drought (seasonal and longer-term)	Pit latrines used as coping mechanism due to water restrictions resulting in faecal contamination of GW	Reduced slow pavement deterioration		Higher risk of blockages in sewer system and discharge pipes	Higher concentration of wastewater leading to less effective treatment
					Higher risk of corrosion of sewers	Corrosive influent damages equipment in treatment plant
		Low moisture content of soil leading to erosion and damage of subsurface structures			Pipe and joint breakages through ground settlement after prolonged drought	Excess deposition due to low flow
					Decreased risk of urban flooding (overflow of inspection chambers, flooding of basements)	
					Decrease in CSO spills	

Climate change effect	Potential hazards and changes	Relevance for urban sanitation failure modes (FM) according to Peal et al. (2020)				
		FM1	FM2	FM3	FM4	FM5
		Faecal sludge not contained not emptied	Faecal sludge and supernatant not delivered to treatment	Faecal sludge and supernatant not treated	Wastewater not delivered to treatment	Wastewater not treated
	Reduced surface water flows	Decreasing levels of hydro-electric productivity resulting in failure of mechanical GW pumps providing water for pour-flush toilets			CSO spills causing higher pollutant concentrations from in receiving waters due to reduced dilution	Less dilution in receiving waters
	Reduced groundwater levels/ resources	Lower GW pollution risk from pit latrines				
Sea-level rise	Rising groundwater levels in coastal/low-lying zones	Higher groundwater/surface water pollution risk caused by increased mobility of pollutants from septic tanks drainfields			Higher groundwater pollution risk	Buoyant forces in areas that were not designed for high GW levels might cause damage to pipes
					Increased dry weather flow in sewer pipe due to infiltration	
						Inflow and infiltration into separate systems causes higher inflow into WWTP that stretch their design capacity
	Saline intrusion in coastal/low-lying zones				Damage to pipes through saltwater infiltration	Saltwater intrusion/inundation affects biological treatment processes
	High water levels (potentially contributing to flooding, erosion, landslides)		Road capacity decreases/Increase in congestions/Travel time increases		WW back-up and flooding through inspection chambers and toilets	Reduced capacity to discharge treated wastewater by gravity/risk of backflow during high tides
			Roads become inaccessible			Damage to WWTP equipment through exposure to saltwater
More variable or increasing temperatures	Higher ambient air temperatures		Damage to road pavements because of degradation of permafrost or other freeze and thaw effects	Increased temperature might increase efficiency of FST in septic tank systems		Moderate increases in temperature might increase efficiency of biological WWT
			Increasing winter temperature reduce pavement damage caused by frost			Variability in winter temperature might lead to deterioration of efficiency of WWT
			More variable winter temperature leading to increase of freeze and thaw events and increasing damage of pavement			
	Hot and cold temperature extremes		Roads become inaccessible because of wildfires			Reduced efficiency of biological treatment if temperature exceeds or falls below operational limits
			Heat damage of (access) roads			

Negative impact	Number of studies
	No original evidence found
	1-3
	4-6
	7-10
	< 10

Positive impact	Number of studies
	No original evidence found
	1-3
	4-6

1. Venkataramanan, V., Packman, A.I., Peters, D.R., Lopez, D., McCuskey, D.J., McDonald, R.I., Miller, W.M. and Young, S.L. A systematic review of the human health and social well-being outcomes of green infrastructure for stormwater and flood management. *Journal of Environmental Management.* 2019, **246**, pp.868-880.
2. Abdellatif, M., Atherton, W. and Alkhaddar, R. Assessing combined sewer overflows with long lead time for better surface water management. *Environmental Technology (United Kingdom).* 2014, **35**(5), pp.568-580.
3. Abdellatif, M., Atherton, W., Alkhaddar, R. and Osman, Y. Application of the UKCP09 WG outputs to assess performance of combined sewers system in a changing climate. *Journal of Hydrologic Engineering.* 2015, **20**(9).
4. Abdellatif, M., Atherton, W., Alkhaddar, R. and Osman, Y. Flood risk assessment for urban water system in a changing climate using artificial neural network. *Natural Hazards.* 2015, **79**(2), pp.1059-1077.
5. Abdellatif, M., Atherton, W., Alkhaddar, R.M. and Osman, Y.Z. Quantitative assessment of sewer overflow performance with climate change in northwest England. *Hydrological Sciences Journal.* 2015, **60**(4), pp.636-650.
6. Abdulla, F. and Farahat, S. Impact of Climate Change on the Performance of Wastewater Treatment Plant: Case study Central Irbid WWTP (Jordan). In: *Procedia Manufacturing*, 2020, pp.205-212.
7. Adin, A., Baumann, E.R. and Warner, F.D. EVALUATION OF TEMPERATURE EFFECTS ON TRICKLING FILTER PLANT PERFORMANCE. *Water Science and Technology.* 1985, **17**(2-3), pp.53-67.
8. Allouche, E.N. Assessment of Damage to Urban Buried Infrastructure in the Aftermath of Hurricanes Katrina and Rita. In: Atalah Alan, T.A. and American Society of Civil Engineers, A.B.D.R.V.A.U.S.A., eds. *Pipelines 2006: Service to the OwnerAmerican Society of Civil Engineers*. Database: Transport Database <1988 to January 2021> Search Strategy:, 2006, p.8.
9. Aralp, C.L., Scheri, J.J. and O'Sullivan, K. Recovering Sandy: Rehabilitation of Wastewater Pumping Stations after Superstorm Sandy. In: *World Environmental And Water Resources Congress 2016: Water, Wastewater, and Stormwater and Urban Watershed Symposium - Papers from Sessions of the Proceedings of the 2016 World Environmental and Water Resources Congress*, 2016, pp.294-302.
10. Bendel, D., Beck, F. and Dittmer, U. Modeling climate change impacts on combined sewer overflow using synthetic precipitation time series. *Water Science and Technology.* 2013, **68**(1), pp.160-166.
11. Budd, E., Babcock, R.W., Jr., Spirandelli, D., Shen, S. and Fung, A. Sensitivity analysis of a groundwater infiltration model and sea-level rise applications for coastal sewers. *Water (Switzerland).* 2020, **12**(3).
12. Budicin, A.N. *Analysis of drought associated impacts on the city of San Bernardino municipal water department's wastewater flow rates and constituent concentrations* Master of Science thesis, California State University - San Bernardino, 2016.
13. Butler, D., McEntee, B., Onof, C. and Hagger, A. Sewer storage tank performance under climate change. *Water science and technology : a journal of the International Association on Water Pollution Research.* 2007, **56**, pp.29-35.
14. Cahoon, L.B. and Hanke, M.H. Inflow and infiltration in coastal wastewater collection systems: Effects of rainfall, temperature, and sea level. *Water Environment Research.* 2019, **91**(4), pp.322-331.
15. Cao, A., Esteban, M. and Mino, T. Adapting wastewater treatment plants to sea level rise: learning from land subsidence in Tohoku, Japan. *Natural Hazards.* 2020, **103**(1), pp.885-902.
16. Chappelle, C., McCann, H., Jassby, D., Schwabe, K. and Szeptycki, L. *Managing Wastewater in a Changing Climate* San Francisco, CA: Public Policy Institute of California, 2019.

17. Danilenko, A., Dickson, E. and Jacobsen, M. *Climate change and urban water utilities : challenges and opportunities.* Washington D.C. : World Bank Group, 2010.

18. Davis, J.A. and Bursztynsky, T.A. Effects of water conservation on municipal wastewater treatment facilities. *Journal of the Water Pollution Control Federation.* 1980, **52**(4), pp.730-739.

19. Draude, S., Keedwell, E., Hiscock, R. and Kapelan, Z. A statistical analysis on the effect of preceding dry weather on sewer blockages in South Wales. *Water Science and Technology.* 2019, **80**(12), pp.2381-2391.

20. Flood, J.F. and Cahoon, L.B. Risks to coastal wastewater collection systems from sea-level rise and climate change. *Journal of Coastal Research.* 2011, **27**(4), pp.652-660.

21. Fortier, C. and Mailhot, A. Climate change impact on combined sewer overflows. *Journal of Water Resources Planning and Management.* 2015, **141**(5).

22. Gamerith, V., Olsson, J., Camhy, D., Hochedlinger, M., Kutschera, P., Schlobinski, S. and Gruber, G. Assessment of Combined Sewer Overflows under Climate Change Urban Drainage Pilot Study Linz. In: *IWA World Congress on Water, Climate and Energy, 2012 May 14-18*, *Dublin, Ireland*. 2012.

23. Gooré Bi, E., Monette, F., Gachon, P., Gaspéri, J. and Perrodin, Y. Quantitative and qualitative assessment of the impact of climate change on a combined sewer overflow and its receiving water body. *Environmental Science and Pollution Research.* 2015, **22**(15), pp.11905-11921.

24. Govere, S., Chikazhe, J. and Mandipa, C.T. Nutrient removal capacities of a domestic wastewater treatment plant under varying rainfall intensities. *Electronic Journal of Environmental, Agricultural and Food Chemistry.* 2010, **9**(10), pp.1619-1630.

25. Hlodversdottir, A.O., Bjornsson, B., Andradottir, H.O., Eliasson, J. and Crochet, P. Assessment of flood hazard in a combined sewer system in Reykjavik city centre. *Water Science and Technology.* 2015, **71**(10), pp.1471-1477.

26. Hwang, J.H. and Oleszkiewicz, J.A. Effect of Cold-Temperature Shock on Nitrification. *Water Environment Research.* 2007, **79**(9), pp.964-968.

27. Kenward, A., Yawitz, D. and Raja, U. *Sewage Overflows From Hurricane Sandy.* Princeton, USA: Climate Central, 2013.

28. Kenward, A., Zenes, N., Bronzan, J., Brady, J. and Shah, K. *Overflow: Climate change, heavy rain, and sewage* Princeton: Climate Central, 2016.

29. Kleidorfer, M., Möderl, M., Sitzenfrei, R., Urich, C. and Rauch, W. A case independent approach on the impact of climate change effects on combined sewer system performance. *Water science and technology : a journal of the International Association on Water Pollution Research.* 2009, **60**, pp.1555-1564.

30. Kleidorfer, M., Mikovits, C., Jasper-Toennies, A., Huttenlau, M., Einfalt, T. and Rauch, W. Impact of a changing environment on drainage system performance. In: Brunone, B.Giustolisi, O.Ferrante, M.Laucelli, D.Meniconi, S.Berardi, L.Campisano, A. eds. *12th International Conference on Computing and Control for the Water Industry, Ccwi2013*. 2014, pp.943-950.

31. Langeveld, J.G., Schilperoort, R.P.S. and Weijers, S.R. Climate change and urban wastewater infrastructure: There is more to explore. *Journal of Hydrology.* 2013, **476**, pp.112-119.

32. Mines Jr, R.O., Lackey, L.W. and Behrend, G.H. The impact of rainfall on flows and loadings at Georgia's wastewater treatment plants. *Water, Air, and Soil Pollution.* 2007, **179**(1-4), pp.135-157.

33. Nie, L., Lindholm, O., Lindholm, G. and Syversen, E. Impacts of climate change on urban drainage systems - a case study in Fredrikstad, Norway. *Urban Water Journal.* 2009, **6**(4), pp.323-332.

34. Nilsen, V., Lier, J.A., Bjerkholt, J.T. and Lindholm, O.G. Analysing urban floods and combined sewer overflows in a changing climate. *Journal of Water and Climate Change.* 2011, **2**(4), pp.260-271.

35. Noi, L.V.T. and Nitivattananon, V. Assessment of vulnerabilities to climate change for urban water and wastewater infrastructure management: Case study in Dong Nai river basin, Vietnam. *Environmental Development.* 2015, **16**, pp.119-137.

36. Pang, Y., Zhang, Y., Yan, X. and Ji, G. Cold Temperature Effects on Long-Term Nitrogen Transformation Pathway in a Tidal Flow Constructed Wetland. *Environmental Science and Technology.* 2015, **49**(22), pp.13550-13557.

37. Plosz, B.G., Liltved, H. and Ratnaweera, H. Climate change impacts on activated sludge wastewater treatment: a case study from Norway. *Water Science and Technology.* 2009, **60**(2), pp.533-541.

38. Rizk, T. and Bhattarai, R.P. Austin water utility wastewater treatment plants flood preparedness, management, and response. In: *87th Annual Water Environment Federation Technical Exhibition and Conference, WEFTEC 2014*, 2014, pp.6505-6529.

39. Sanders, D.A. Damage to wastewater treatment facilities from Great Flood of 1993. *Journal of Environmental Engineering.* 1997, **123**(1), pp.54-60.

40. Semadeni-Davies, A., Hernebring, C., Svensson, G. and Gustafsson, L.-G. The impacts of climate change and urbanisation on drainage in Helsingborg, Sweden: Combined sewer system. *Journal of Hydrology.* 2008, **350**(1-2), pp.100-113.

41. Shorney, F.L. Impacts and lessons learned from the flood of 1993. *Public Works.* 1994, **125**(7), pp.38-40.

42. Tait, S.J., Ashley, R.M., Cashman, A., Blanksby, J. and Saul, A.J. Sewer system operation into the 21st century, study of selected responses from a UK perspective. *Urban Water Journal.* 2008, **5**(1), pp.79-88.

43. Takamatsu, M., Nakazato, T., Fischer, R., Satoh, H., Bonaccorso, F. and Grey, G. Climatological disasters and their impact on wastewater treatment infrastructure - A comparison of Japan's tsunami and superstorm Sandy STP damage, response, and mitigation. In: *87th Annual Water Environment Federation Technical Exhibition and Conference, WEFTEC 2014*, 2014, pp.1250-1261.

44. Teichmann, M., Szeligova, N. and Kuda, F. Influence of flash floods on the drainage systems of the urbanized areas. In: *Advances and Trends in Engineering Sciences and Technologies III- Proceedings of the 3rd International Conference on Engineering Sciences and Technologies, ESaT 2018*, 2019, pp.623-628.

45. The World Bank. Resilient water supply and sanitation services: the case of Japan. *Resilient water supply and sanitation services: the case of Japan.* 2018, pp.viii-pp.

46. Wood, D.M., Roche, A., Chokshi, M. and May, K. Preparing the San Francisco sewer system for the looming compound challenge of climate change induced rainfall, extreme tides, and sea level rise. In: *88th Annual Water Environment Federation Technical Exhibition and Conference, WEFTEC 2015*, 2015, pp.6399-6415.

47. Zamanian, S., Rahimi, M. and Shafieezadeh, A. Resilience of Sewer Networks to Extreme Weather Hazards: Past Experiences and an Assessment Framework. In: *Pipelines 2020: Utility Engineering, Surveying, and Multidisciplinary Topics - Proceedings of Sessions of the Pipelines 2020 Conference*, 2020, pp.50-59.

48. Cooper, J.A., Loomis, G.W. and Amador, J.A. Hell and high water: Diminished septic system performance in coastal regions due to climate change. *PLoS ONE.* 2016, **11**(9).

49. Heath, T.T., Parker, A.H. and Weatherhead, E.K. Testing a rapid climate change adaptation assessment for water and sanitation providers in informal settlements in three cities in sub-Saharan Africa. *Environment and Urbanization.* 2012, **24**(2), pp.619-637.

50. Hoque, B.A., Huttly, S.R.A., Aziz, K.M.A., Hasan, Z. and Patwary, M.Y. Effects of Floods on the Use and Condition of Pit Latrines in Rural Bangladesh. *Disasters.* 1989, **13**(4), pp.315-321.

51. Kazi, M.N. and Rahman, M.M. Sanitation strategies for flood-prone areas. In: *25th WEDC Conference: Integrated Development for Water Supply and Sanitation*, *Addis Ababa, Ethiopia*. 1999.

52. Morales, I., Amador, J.A. and Boving, T. Bacteria transport in a soil-based wastewater treatment system under simulated operational and climate change conditions. *Journal of Environmental Quality.* 2015, **44**(5), pp.1-14.

53. O'Driscoll, M.A., Humphrey, C.P., Jr., Deal, N.E., Lindbo, D.L. and Zarate-Bermudez, M.A. Meteorological influences on nitrogen dynamics of a coastal onsite wastewater treatment system. *Journal of Environmental Quality.* 2014, **43**(6), pp.1873-1885.

54. Shimi, A.C., Parvin, G.A., Biswas, C. and Shaw, R. Impact and adaptation to flood: A focus on water supply, sanitation and health problems of rural community in Bangladesh. *Disaster Prevention and Management: An International Journal.* 2010, **19**(3), pp.298-313.

55. Viraraghavan, T. INFLUENCE OF TEMPERATURE ON PERFORMANCE OF SEPTIC TANK SYSTEMS. *Water Air and Soil Pollution.* 1977, **7**(1), pp.103-110.

56. Alfaro Marolo, C., Ciro German, A., Thiessen Kendall, J. and Ng, T. Case Study of Degrading Permafrost Beneath a Road Embankment. *Journal of Cold Regions Engineering.* 2009, **23**, pp.93-111.

57. Alhassan, H.M. and Ben-Edigbe, J. Effect of rainfall intensity variability on highway capacity. *European Journal of Scientific Research.* 2011, **49**(1), pp.18-27.

58. Anarde, K.A., Kameshwar, S., Irza, J.N., Nittrouer, J.A., Lorenzo-Trueba, J., Padgett, J.E., Sebastian, A. and Bedient, P.B. Impacts of Hurricane Storm Surge on Infrastructure Vulnerability for an Evolving Coastal Landscape. *Natural Hazards Review.* 2018, **19**(1).

59. Aursand, P.O., Evensen, R. and Lerfald, B.O. Climate changes in Norway: factors affecting pavement performance. In: *Ninth International Conference on the Bearing Capacity of Roads, Railways and Airfields*, Database: Transport Database <1988 to January 2021> Search Strategy:: Akademika Publishing, 2013, pp.537-544.

60. Balakrishnan, S., Zhang, Z., Machemehl, R. and Murphy, M.R. Mapping resilience of Houston freeway network during Hurricane Harvey using extreme travel time metrics. *International Journal of Disaster Risk Reduction.* 2020, **47**.

61. Bíl, M., Vodák, R., Kubeček, J., Bílová, M. and Sedoník, J. Evaluating road network damage caused by natural disasters in the Czech Republic between 1997 and 2010. *Transportation Research Part A: Policy and Practice.* 2015, **80**, pp.90-103.

62. Bilodeau, J.P., Drolet, F.P., Doré, G. and Sottile, M.F. Effect of Climate Changes Expected during Winter on Pavement Performance. In: *Proceedings of the International Conference on Cold Regions Engineering*, 2015, pp.617-628.

63. Bucar, R.C.B. and Hayeri, Y.M. Quantitative assessment of the impacts of disruptive precipitation on surface transportation. *Reliability Engineering and System Safety.* 2020, **203**.

64. Chang, H., Lafrenz, M., Jung Il, W., Figliozzi, M. and Platman, D. Potential Impacts of Climate Change on Urban Flooding: Implications for Transportation Infrastructure and Travel Disruption. In: *2009 International Conference on Ecology and Transportation (ICOET 2009), Duluth, Minnesota*. Raleigh N. C. United States: Center for Transportation and the Environment, North Carolina State University, 2009, pp.72-79.

65. Chang, H., Lafrenz, M., Jung, I.-W., Figliozzi, M., Platman, D. and Pederson, C. Potential Impacts of Climate Change on Flood-Induced Travel Disruptions: A Case Study of Portland, Oregon, USA. *Annals of the Association of American Geographers.* 2010, **100**(4), pp.938-952.

66. Chen, X. and Zhang, Z. Effects of hurricanes katrina and rita flooding on louisiana pavement performance. In: *Geotechnical Special Publication*, 2014, pp.212-221.

67. Diakakis, M., Boufidis, N., Salanova Grau, J.M., Andreadakis, E. and Stamos, I. A systematic assessment of the effects of extreme flash floods on transportation infrastructure and circulation: The example of the 2017 Mandra flood. *International Journal of Disaster Risk Reduction.* 2020, **47**.

68. Ebuzoeme and Divine-Favour, O. Evaluating the effects of flooding in six communities in Awka Anambra state of Nigeria. *Journal of Environment and Earth Science.* 2015, **5**(4), pp.26-38.

69. Elshaer, M., Ghayoomi, M. and Daniel, J.S. Impact of subsurface water on structural performance of inundated flexible pavements. *International Journal of Pavement Engineering.* 2019, **20**(8), pp.947-957.

70. Friedrich, E. and Timol, S. Climate change and urban road transport - A South African case study of vulnerability due to sea level rise. *Journal of the South African Institution of Civil Engineering.* 2011, **53**(2), pp.14-22.

71. Hemed, A., Ouadif, L., Bahi, L. and Lahmili, A. Impact of climate change on pavements. In: *E3S Web of Conferences*, 2020.

72. Ismail, M.S.N. and Ghani, A.N.A. An Overview of Road Damages Due to Flooding: Case Study in Kedah State, Malaysia. In: Aziz, H.A.AbuBakar, B.H.Johari, M.A.M.Keong, C.K.Yusoff, M.S.Hasan, M.R.M.Ramli, M.H.Halim, H. eds. *Proceedings of the International Conference of Global Network for Innovative Technology and Awam International Conference in Civil Engineering*. 2017.

73. Jacobs, J.M., Cattaneo, L.R., Sweet, W. and Mansfield, T. Recent and Future Outlooks for Nuisance Flooding Impacts on Roadways on the US East Coast. *Transportation Research Record.* 2018, **2672**(2), pp.1-10.

74. Lu, Q.-C. and Peng, Z.-R. Vulnerability Analysis of Transportation Network Under Scenarios of Sea Level Rise. *Transportation Research Record.* 2011, (2263), pp.174-181.

75. Mallick, R.B., Jacobs, J.M., Miller, B.J., Daniel, J.S. and Kirshen, P. Understanding the impact of climate change on pavements with CMIP5, system dynamics and simulation. *International Journal of Pavement Engineering.* 2018, **19**(8), pp.697-705.

76. Mallick, R.B., Radzicki, M.J., Daniel, J.S. and Jacobs, J.M. Use of System Dynamics to Understand Long-Term Impact of Climate Change on Pavement Performance and Maintenance Cost. *Transportation Research Record.* 2014, (2455), pp.1-9.

77. Mills, B.N., Tighe, S.L., Andrey, J., Smith, J.T. and Huen, K. Climate change implications for flexible pavement design and performance in Southern Canada. *Journal of Transportation Engineering.* 2009, **135**(10), pp.773-782.

78. Mitsakis, E., Stamos, I., Diakakis, M. and Grau, J.M.S. Impacts of high-intensity storms on urban transportation: applying traffic flow control methodologies for quantifying the effects. *International Journal of Environmental Science and Technology.* 2014, **11**(8), pp.2145-2154.

79. Mitsakis, E., Stamos, I., Papanikolaou, A., Aifadopoulou, G. and Kontoes, H. Assessment of extreme weather events on transport networks: case study of the 2007 wildfires in Peloponnesus. *Natural Hazards.* 2014, **72**(1), pp.87-107.

80. Mndawe, M.B., Ndambuki, J.M., Kupolati, W.K., Badejo, A.A. and Dunbar, R. Assessment of the effects of climate change on the performance of pavement subgrade. *African Journal of Science, Technology, Innovation and Development.* 2015, **7**(2), pp.111-115.

81. Mosqueda, G., Porter, K.A., O'Connor, J. and McAnany, P. Damage to engineered buildings and bridges in the wake of hurricane Katrina. In: *Forensic Engineering (2007)*, 2007.

82. Praharaj, S., Chen, T.D., Zahura, F.T., Behl, M. and Goodall, J.L. Estimating impacts of recurring flooding on roadway networks: a Norfolk, Virginia case study. *Natural Hazards.* 2021.

83. Pyatkova, K., Chen, A.S., Butler, D., Vojinović, Z. and Djordjević, S. Assessing the knock-on effects of flooding on road transportation. *Journal of Environmental Management.* 2019, **244**, pp.48-60.

84. Qiao, Y., Santos, J., Stoner, A.M.K. and Flinstch, G. Climate change impacts on asphalt road pavement construction and maintenance: An economic life cycle assessment of adaptation measures in the State of Virginia, United States. *Journal of Industrial Ecology.* 2020, **24**(2), pp.342-355.

85. Read, W. and Reed, D. The 2006 hanukkah eve storm and associated civil infrastructure damage in the Cascadia region of the United States and Canada. In: *12th Americas Conference on Wind Engineering 2013, ACWE 2013: Wind Effects on Structures, Communities, and Energy Generation*, 2013, pp.1296-1315.

86. Shao, Z., Jenkins, G. and Oh, E. Assessing the impacts of climate change on road infrastructure. *International Journal of GEOMATE.* 2017, **13**(38), pp.120-128.

87. Stoner, A.M.K., Daniel, J.S., Jacobs, J.M., Hayhoe, K. and Scott-Fleming, I. Quantifying the Impact of Climate Change on Flexible Pavement Performance and Lifetime in the United States. *Transportation Research Record.* 2019, **2673**(1), pp.110-122.

88. Suarez, P., Anderson, W., Mahal, V. and Lakshmanan, T.R. Impacts of flooding and climate change on urban transportation: A systemwide performance assessment of the Boston Metro Area. *Transportation Research Part D: Transport and Environment.* 2005, **10**(3), pp.231-244.

89. Sultana, M., Chai, G., Chowdhury, S. and Martin, T. Deterioration of flood affected Queensland roads – An investigative study. *International Journal of Pavement Research and Technology.* 2016, **9**(6), pp.424-435.

90. Sultana, M., Chai, G., Chowdhury, S. and Martin, T. Rapid Deterioration of Pavements Due to Flooding Events in Australia. In: *Geotechnical Special Publication*, 2016, pp.104-112.

91. Sultana, M., Chowdhury, S., Chai, G. and Martin, T. Modelling rapid deterioration of flooded pavements. *Road and Transport Research.* 2016, **25**(2), pp.3-14.

92. Thiam, P.M., Dore, G. and Bilodeau, J.P. Effect of the future increases of precipitation on the long-term performance of roads. In: *Ninth International Conference on the Bearing Capacity of Roads, Railways and Airfields*, *Trondheim, Norway* Akademika, Publishing, 2013, pp.545-554.

93. Tighe, S.L., Smith, J., Mills, B. and Andrey, J. Evaluating Climate Change Impact an Low-Volume Roads in Southern Canada. *Transportation Research Record.* 2008, (2053), pp.9-16.

94. Ying, L.K. and Abdul Ghani, A.N. Rainfall characteristics and its effect on road infrastructure health. *International Journal of Integrated Engineering.* 2019, **11**(9 Special Issue), pp.234-246.

95. Zhang, Z., Wu, Z., Martinez, M. and Gaspard, K. Pavement structures damage caused by Hurricane Katrina flooding. *Journal of Geotechnical and Geoenvironmental Engineering.* 2008, **134**(5), pp.633-643.

96. Alam, S.S., Alam, A.J. and Rahman, S. *Urban climate resilience, water and sanitation: improving multi-stakeholder collaboration in Dhaka, Bangladesh.* London, 2015.

97. Howard, G. and Bartram, J. *Vision 2030: The resilience of water supply and sanitation in the face of climate change. Technical Report.* Geneva: World Health Organisation, 2010.

98. Clemenz, N., Boakye, R. and Parker, A. Rapid climate adaption assessment (Rcaa) of water supply and sanitation services in two coastal urban poor communities in accra, ghana. *Journal of Water and Climate Change.* 2020, **11**(4), pp.1645-1660.

99. McGill, B.M., Altchenko, Y., Hamilton, S.K., Kenabatho, P.K., Sylvester, S.R. and Villholth, K.G. Complex interactions between climate change, sanitation, and groundwater quality: a case study from Ramotswa, Botswana. *Hydrogeology Journal.* 2019, **27**(3), pp.997-1015.

100. Purwar, D., Sliuzas, R. and Flacke, J. Assessment of cascading effects of typhoons on water and sanitation services: A case study of informal settlements in Malabon, Philippines. *International Journal of Disaster Risk Reduction.* 2020, **51**.
